# Mental Health Conditions in Partners and Adult Children of Stroke Survivors

**DOI:** 10.1001/jamanetworkopen.2024.3286

**Published:** 2024-03-14

**Authors:** Nils Skajaa, Dóra Körmendiné Farkas, Kristina Laugesen, Cecilia Hvitfeldt Fuglsang, Victor W. Henderson, Oleguer Plana-Ripoll, David Gaist, Henrik Toft Sørensen

**Affiliations:** 1 Department of Clinical Epidemiology, Department of Clinical Medicine, Aarhus University, Aarhus University Hospital, Aarhus, Denmark; 2 Department of Epidemiology & Population Health, Stanford University, Stanford, California; 3 Department of Neurology & Neurological Sciences, Stanford University, Stanford, California; 4 Research Unit for Neurology, Odense University Hospital and University of Southern Denmark, Odense, Denmark

## Abstract

**Importance:**

Family caregiving after critical illness has been associated with several adverse health outcomes, including various aspects of mental health, but research focusing specifically on family members of stroke survivors is limited.

**Objectives:**

To examine the associations of stroke in a partner or parent with the risk of depression, substance use disorders, anxiety disorders, and self-harm or suicide.

**Design, Setting, and Participants:**

This nationwide, population-based cohort study used data from Danish nationwide administrative and clinical registries (2004-2021). Participants included partners and adult children of survivors of stroke. Data analysis was performed from March to December 2023.

**Exposure:**

Having a partner or parent who survived stroke.

**Main Outcomes and Measures:**

The Aalen-Johansen estimator was used to compute propensity score–weighted 3-year absolute risks, risk differences, and risk ratios for depression, substance use disorders, anxiety disorders, and self-harm or suicide among partners or children of survivors of stroke compared with partners or children of survivors of myocardial infarction (MI) and matched individuals from the general population.

**Results:**

The study included a total of 1 923 732 individuals: 70 917 partners of stroke survivors (median [IQR] age, 68 [59-76] years; 46 369 women [65%]), 70 664 partners of MI survivors (median [IQR] age, 65 [55-73] years; 51 849 women [73%]), 354 570 partners of individuals from the general population (median [IQR] age, 68 [59-76] years; 231 833 women [65%]), 207 386 adult children of stroke survivors (median [IQR] age, 45 [36-52] years; 99 382 women [48%]), 183 309 adult children of MI survivors (median [IQR] age, 42 [33-49] years; 88 078 women [48%]), and 1 036 886 adult children of individuals from the general population (median [IQR] age, 45 [36-52] years; 496 875 women [48%]). Baseline characteristics were well balanced across cohorts after propensity score weighting. Among partners of stroke survivors, the 3-year absolute risk was 1.0% for depression, 0.7% for substance use disorders, 0.3% for anxiety disorders, and 0.04% for self-harm or suicide. Risk ratio point estimates for the assessed outcomes ranged from 1.14 to 1.42 compared with the general population and from 1.04 to 1.09 compared with partners of MI survivors. The elevated risk of depression in partners of stroke survivors was more pronounced after severe or moderate stroke than after mild stroke. Among adult children of stroke survivors, the 3-year absolute risk was 0.6% for depression, 0.6% for substance use disorders, 0.2% for anxiety disorders, and 0.05% for self-harm or suicide. Both absolute risks and risk ratios for adult children of stroke survivors were smaller than those reported in the partner analyses.

**Conclusions and Relevance:**

In this cohort study of partners and adult children of stroke survivors, risks of several mental health conditions and self-harm or suicide were moderately higher compared with the general population and, to a lesser extent, partners and adult children of MI survivors. These findings highlight the potential consequences of stroke among family members, particularly partners, and its findings may possibly serve as a quantitative foundation for the development of future stroke rehabilitation services.

## Key Points

**Question:**

Are partners and adult children of stroke survivors at increased risk of depression, substance use disorders, anxiety disorders, and self-harm or suicide?

**Findings:**

In this cohort study or more than 1.9 million individuals, family members, particularly partners, of stroke survivors had moderately higher risks of several mental health conditions and self-harm or suicide compared with the Danish general population and, to a lesser extent, family members of myocardial infarction survivors.

**Meaning:**

These findings highlight the potential mental health consequences of stroke among family members and may possibly serve as a quantitative foundation for the development of future stroke rehabilitation services.

## Teaser

This cohort study examines the association of stroke in a partner or parent with the risk of depression, substance use disorders, anxiety disorders, and self-harm or suicide among participants in Denmark.

## Introduction

Stroke is common, disabling, and costly.^1,2^ Survival after stroke has improved dramatically in recent decades in most geographic settings, including Denmark,^1,3^ which has resulted in a surge in the absolute number of stroke survivors.

After surviving a stroke, patients often cope with persistent disability. Subsequent cardiovascular events and neurological and mental health complications are also common in these patients,^4,5,6^ underscoring the challenges faced by patients and their families. Indeed, family members of stroke survivors may assume essential roles in the stroke recovery process, often acting as informal caregivers.^7^ Caring for loved ones after illness can be grueling and may lead to chronic stress, fatigue, social isolation, and deleterious behavioral changes (eg, less exercise).^8,9^ Other studies have found an association between caregiving and adverse health outcomes, most prominently psychological distress^10^ (including suicidal ideation^11^), cardiovascular disease,^9,12^ and premature mortality.^13^

Research focusing on mental health aspects of family members or caregivers specifically after stroke is limited.^14,15,16,17,18,19,20,21,22^ Moreover, most earlier studies were small (<500 individuals)^14,15,16,17,18,20,21^ and lacked longitudinal data based on a population-based study design with relevant comparison cohorts^14,15,16,17,18,19,20,21,22^ (an overview of the existing literature is provided in eTable 1 in Supplement 1). To advance this body of research, the inclusion of relevant comparison cohorts remains essential. In this nationwide, population-based study, we (1) examine the associations of stroke in a partner or parent with depression, substance use disorders, anxiety disorders, self-harm or suicide, and a composite mental health condition outcome; (2) provide context to these risk estimates using general population (GP) (to fully understand the magnitude of effect estimates) and myocardial infarction (MI) (as an active comparator^23,24^) comparison cohorts; and (3) explore the extent to which stroke severity, stroke subtype, age, sex, comorbidity, and socioeconomic position may moderate these associations.

## Methods

### Data Sources

This cohort study used data from Danish clinical and administrative registries with long-term, nationwide coverage.^25^ Data sources (described in detail in the eAppendix in Supplement 1) were linked at the individual level using a unique personal identification number assigned to each resident.^26^ The cumulative source population during the study period (2004-2021) was 6 157 156 individuals. All codes and definitions are listed in eTable 2 in Supplement 1. We followed the Strengthening the Reporting of Observational Studies in Epidemiology (STROBE) reporting guidelines for cohort studies. Ethical approval and informed consent of study participants are not required for registry-based studies under Danish law. The study was approved by the Danish Data Protection Agency at Aarhus University.

### Setting and Stroke Management

In Denmark, health care is tax financed and, therefore, free of personal charge for all residents. Stroke management in Denmark is detailed in the eAppendix in Supplement 1. In brief, specialized stroke rehabilitation is initiated during in-hospital care; after hospital discharge, rehabilitation is managed at the municipal level, and few data exist regarding its implementation and effectiveness.^27^

### Study Cohorts and Follow-Up

We assembled 2 exposed cohorts: partners of stroke survivors (ie, the stroke-partner cohort) and adult children of stroke survivors (ie, the stroke-offspring cohort) (eFigure 1 in Supplement 1). From the Danish Stroke Registry,^28^ we identified patients (aged ≥18 years) hospitalized with a first-time stroke (ischemic stroke or intracerebral hemorrhage) between May 1, 2004, and December 31, 2021, who were discharged from hospital alive. Using linked information in the Civil Registration System,^26^ we identified stroke survivors’ partners and adult children. Partners were defined as follows: (1) 2 people who were married (including civil unions of same-sex couples), (2) 2 people who were cohabitating with a shared child, or (3) 2 cohabitating individuals of opposite sex, with an age difference less than 15 years and without a shared child. Both biological and adopted children were eligible for inclusion. We then assembled 2 unexposed comparison cohorts for each of the 2 exposed cohorts: partners and adult children of individuals from the GP without stroke or MI (GP-partner cohort and GP-offspring cohort) and partners and adult children of MI survivors (MI-partner cohort and MI-offspring cohort).

In all cohorts, we excluded individuals with a diagnosis of a mental health condition before the index date (defined as the partner’s or parent’s stroke discharge date for the stroke-partner and stroke-offspring cohorts, the matched partner’s or parent’s stroke discharge date for the GP-partner and GP-offspring cohorts, and the partner’s or parent’s MI discharge date for the MI-partner and MI-offspring cohorts) and those younger than 18 years. A full description of how cohorts were constructed is provided in the eAppendix in Supplement 1. Follow-up for all cohort members began on the index date and continued for up to 3 years (data were available until the end of 2021) or until the occurrence of an outcome, death, or emigration, whichever occurred first.

### Mental Health Conditions and Self-Harm or Suicide

We defined 4 outcomes with high clinical relevance and mechanistic plausibility^8^: (1) depression (*International Statistical Classification of Diseases and Related Health Problems, Tenth Revision* [*ICD-10*] codes F32-F33), (2) substance use disorders (*ICD-10* codes F10-F19), (3) anxiety disorders (*ICD-10* code F41), and (4) intentional self-harm or suicide (ie, suicide attempts or completions; *ICD-10* codes X60-X84). As a fifth outcome, to facilitate a broad understanding of the overall mental health burden, we defined a composite outcome as the first occurrence of a hospital diagnosis of any mental health condition (*ICD-10* codes F00-F99 and G30). Self-harm or suicide was not included in the composite outcome definition. Mental health conditions were identified from hospital-based diagnoses in the Patient Registry^29^ or the Psychiatric Central Research Database^30^; self-harm or suicide was identified from hospital-based diagnoses or cause of death records in the Registry of Causes of Death^31^ (see eTable 2 in Supplement 1 for precise definitions).

### Baseline Characteristics

On the basis of the causal assumptions depicted in eFigure 2 in Supplement 1, the following baseline characteristics, measured before the index date, were selected as potential confounders: demographics, year of index date, household income, highest achieved education, comorbidity, comedications, and health care utilization (see eAppendix and eTable 2 in Supplement 1 for definitions). In the stroke-partner and stroke-offspring cohorts only, we further obtained information on stroke severity at time of admission, as measured by the Scandinavian Stroke Scale (mild, 43-58; moderate, 26-42; or severe, 0-25)^32^ and stroke subtype (ischemic stroke or intracerebral hemorrhage).

### Statistical Analysis

Data analysis was performed from March to December 2023. We used propensity score (PS) standardized morbidity ratio weighting to control for baseline imbalances between cohorts (eAppendix in Supplement 1).^33^ This weighting approach reweighted the unexposed cohorts so that their covariate distribution resembled that in the exposed cohorts. Missing data on income (0.2%) and education (1.9%) were handled using a missing data indicator variable in the PS estimation.^34^ We assessed covariate balance after weighting using standardized mean differences, with values less than 0.1 indicative of balance.^35^

We used the Aalen-Johansen estimator, which accounts for death as a competing event,^36^ to calculate weighted 3-year absolute risks, risk differences (RDs), and risk ratios (RRs) comparing the stroke-partner and stroke-offspring cohorts with the GP-partner and GP-offspring and MI-partner and MI-offspring cohorts. Corresponding 95% CIs were obtained through nonparametric bootstrapping using 200 resamples.^37^

We stratified the analyses according to stroke severity, stroke subtype, age group, sex, number of baseline comorbidities, household income, and highest achieved education. PS weights were re-estimated within each examined stratum.^38^

In sensitivity analyses, we (1) altered the depression definition also incorporating filled prescriptions, (2) altered the depression definition also incorporating persistent mood disorders (*ICD-10* codes F32-F34), (3) used nonmelanoma skin cancer as a negative control outcome, (4) performed a complete-case analysis, and (5) set the index date as the stroke admission date instead of the discharge date (eAppendix in Supplement 1). Statistical analysis was performed with SAS statistical software version 9.4 (SAS Institute), and data visualization was performed with RStudio software version 2023.06.01 (mounting R version 4.3.1; R Project for Statistical Computing).

## Results

### Partners of Stroke Survivors

#### Cohort Characteristics

A total of 1 923 732 individuals were included in the cohorts. The partner cohorts consisted of 70 917 partners of stroke survivors (91% ischemic strokes, 72% of mild severity; median [IQR] age, 68 [59-76] years; 46 369 women [65%]), 354 570 partners of individuals in the GP (median [IQR] age, 68 [59-76] years; 231 833 women [65%]), and 70 664 partners of MI survivors (median [IQR] age, 65 [55-73] years; 51 849 women [73%]) (eTable 3 in Supplement 1). Even before PS weighting, baseline characteristics were relatively well balanced across cohorts. The proportion with a high educational level (International Standard Classification of Education level 5-8) was 20% among partners of stroke survivors, 25% among partners in the GP, and 20% among partners of MI survivors. There were no clear differences across cohorts in health care utilization in the prior 3 years (eTable 3 in Supplement 1). After PS weighting, virtually all baseline characteristics had standardized mean differences less than 0.01. The RDs and RRs presented later in this article are PS weighted.

#### Main Analyses

**Figure 1.**
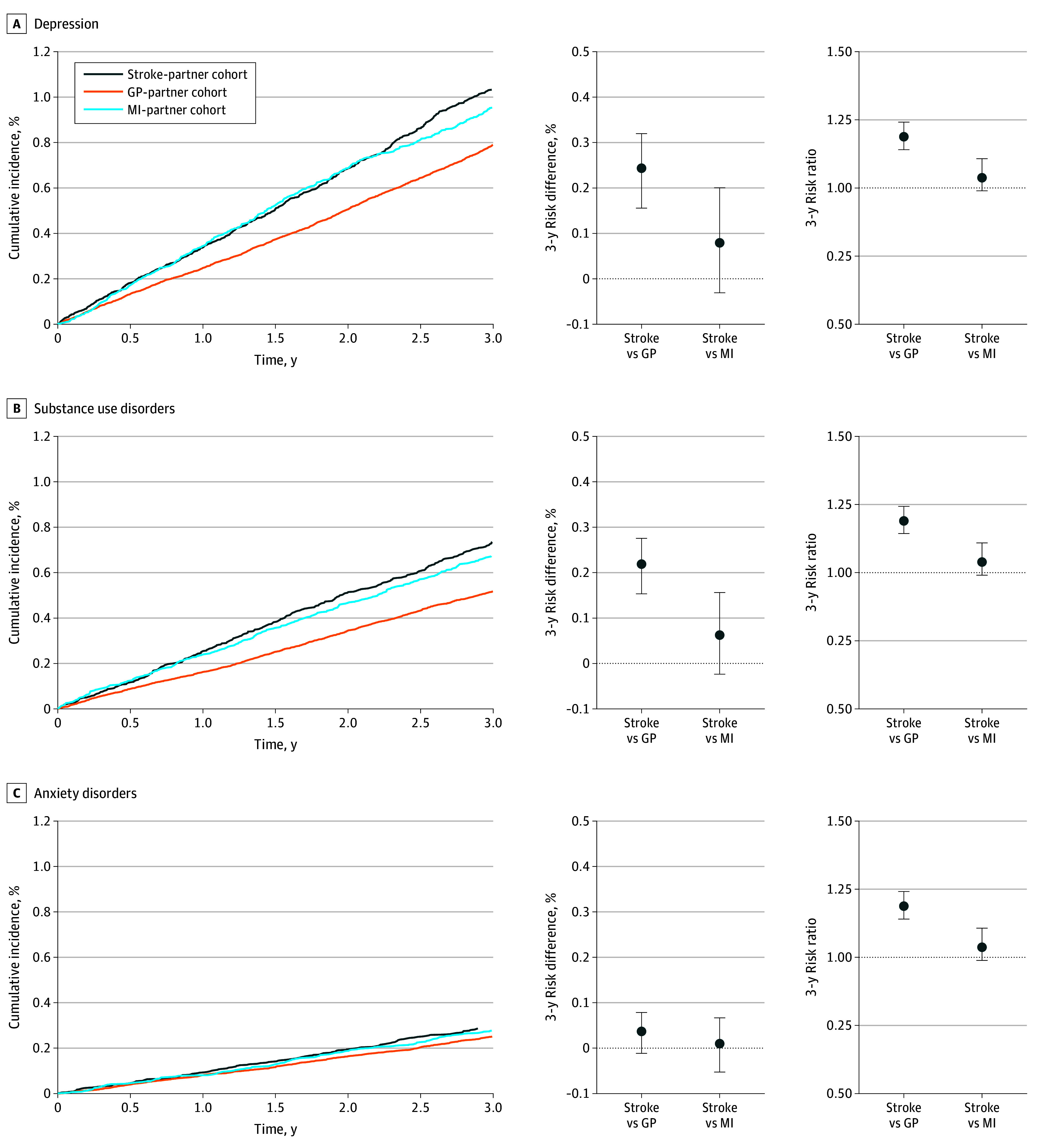
Risk of Mental Health Conditions Among Partners of Individuals With Stroke or Myocardial Infarction (MI) and Among the General Population Graphs show propensity score–weighted cumulative incidences, 3-year risk differences, and 3-year risk ratios of depression (A), substance use disorders (B), and anxiety disorders (C) among partners of stroke survivors (stroke-partner cohort), partners of individuals from the general population (GP-partner cohort), and partners of MI survivors (MI-partner cohort). Error bars denote 95% CIs.

Among partners of stroke survivors, the 3-year absolute risk was 1.0% for depression, 0.7% for substance use disorders, 0.3% for anxiety disorders, 0.04% for self-harm or suicide, and 4.1% for the composite outcome of any diagnosis of a mental health condition (Figure 1 and eTable 4 and eFigure 3 in Supplement 1). Compared with the GP-partner cohort, the RDs and RRs were 0.24% (95% CI, 0.16% to 0.32%) and 1.31 (95% CI, 1.19 to 1.41) for depression, 0.22% (95% CI, 0.15% to 0.28%) and 1.42 (95% CI, 1.29 to 1.55) for substance use disorders, 0.04% (95% CI, −0.01% to 0.08%) and 1.14 (95% CI, 0.95 to 1.33) for anxiety disorders, 0.01% (95% CI, −0.01% to 0.03%) and 1.25 (95% CI, 0.76 to 1.94) for self-harm or suicide, and 0.64% (95% CI, 0.48% to 0.82%) and 1.19 (95% CI, 1.14 to 1.24) for the composite outcome (Table 1, Figure 1, and eFigure 3 in Supplement 1). Compared with the MI-partner cohort, the RDs and RRs were 0.08% (95% CI, −0.03% to 0.20%) and 1.08 (95% CI, 0.97 to 1.23) for depression, 0.06% (95% CI, −0.02% to 0.16%) and 1.09 (95% CI, 0.97 to 1.25) for substance use disorders, 0.01% (95% CI, −0.05% to 0.07%) and 1.04 (95% CI, 0.83 to 1.27) for anxiety disorders, 0.00% (95% CI, −0.02% to 0.02%) and 1.09 (95% CI, 0.60 to 2.04) for self-harm or suicide, and 0.15% (95% CI −0.04% to 0.40%) and 1.04 (95% CI, 0.99 to 1.11) for the composite outcome (Table 1, Figure 1, and eFigure 3 in Supplement 1).

**Table 1.** Associations of Stroke in a Partner or Parent With Risk of Depression, Substance Use Disorders, Anxiety Disorders, Self-Harm or Suicide, and a Composite Outcome of Any Diagnosis of an MHC

Cohort and outcome	Stroke vs GP		Stroke vs MI	
	3-y RD, % (95% CI)^a^	3-y RR (95% CI)^a^	3-y RD, % (95% CI)^a^	3-y RR (95% CI)^a^
Partner cohort				
Depression	0.24 (0.16 to 0.32)	1.31 (1.19 to 1.41)	0.08 (−0.03 to 0.20)	1.08 (0.97 to 1.23)
Substance use disorders	0.22 (0.15 to 0.28)	1.42 (1.29 to 1.55)	0.06 (−0.02 to 0.16)	1.09 (0.97 to 1.25)
Anxiety disorders	0.04 (−0.01 to 0.08)	1.14 (0.95 to 1.33)	0.01 (−0.05 to 0.07)	1.04 (0.83 to 1.27)
Self-harm or suicide^b^	0.01 (−0.01 to 0.03)	1.25 (0.76 to 1.94)	0.00 (−0.02 to 0.02)	1.09 (0.60 to 2.04)
Any diagnosis of MHC	0.64 (0.48 to 0.82)	1.19 (1.14 to 1.24)	0.15 (−0.04 to 0.40)	1.04 (0.99 to 1.11)
Offspring cohort				
Depression	0.04 (−0.00 to 0.07)	1.07 (0.99 to 1.14)	−0.00 (−0.06 to 0.05)	0.99 (0.90 to 1.09)
Substance use disorders	0.06 (0.03 to 0.10)	1.11 (1.05 to 1.19)	−0.02 (−0.07 to 0.04)	0.97 (0.89 to 1.06)
Anxiety disorders	0.02 (−0.01 to 0.05)	1.10 (0.97 to 1.25)	0.01 (−0.02 to 0.04)	1.05 (0.90 to 1.22)
Self-harm or suicide^b^	0.01 (0.00 to 0.03)	1.42 (1.11 to 1.84)	0.01 (−0.00 to 0.02)	1.28 (0.91 to 1.77)
Any diagnosis of MHC	0.17 (0.10 to 0.24)	1.09 (1.05 to 1.12)	−0.05 (−0.14 to 0.04)	0.98 (0.94 to 1.02)

Abbreviations: GP, general population; MHC, mental health condition; MI, myocardial infarction; RD, risk difference; RR, risk ratio.

^a^ RDs and RRs were weighted for the following variables: age and sex of both study participants and their respective partners or parents, year of index date, household income, highest achieved education, comorbidities (32 distinct conditions), comedications, and health care utilization (full list in eTable 2 in Supplement 1).

^b^ Self-harm or suicide was not included in the composite outcome of any MHC.

#### Subgroup Analyses

Stroke severity modified the association with depression (Table 2). Compared with the GP-partner cohort, the RRs in the stroke-partner cohort were 1.26 (95% CI, 1.14-1.38) for mild stroke, 1.34 (95% CI, 1.11-1.61) for moderate stroke, and 1.46 (95% CI, 1.13-1.73) for severe stroke. Compared with the MI-partner cohort, the RRs in the stroke-partner cohort were 1.04 (95% CI, 0.90-1.15) for mild stroke, 1.12 (95% CI, 0.91-1.37) for moderate stroke, and 1.21 (95% CI, 0.95-1.49) for severe stroke. Additional subgroup results are presented in eTables 5 to 10 in Supplement 1.

**Table 2.** Associations of Stroke in a Partner or Parent With Risk of Depression, Substance Use Disorders, Anxiety Disorders, Self-Harm or Suicide, and a Composite Outcome of Any Diagnosis of an MHC, Stratified by Stroke Severity

Cohort, outcome, and stroke severity	Stroke vs GP		Stroke vs MI	
	3-y RD, % (95% CI)^a^	3-y RR (95% CI)^a^	3-y RD, % (95% CI)^a^	3-y RR (95% CI)^a^
Partner cohort				
Depression				
Mild	0.19 (0.10 to 0.28)	1.26 (1.14 to 1.38)	0.03 (−0.10 to 0.13)	1.04 (0.90 to 1.15)
Moderate	0.31 (0.10 to 0.54)	1.34 (1.11 to 1.61)	0.13 (−0.10 to 0.38)	1.12 (0.91 to 1.37)
Severe	0.41 (0.12 to 0.64)	1.46 (1.13 to 1.73)	0.23 (−0.06 to 0.50)	1.21 (0.95 to 1.49)
Substance use disorders				
Mild	0.22 (0.14 to 0.31)	1.45 (1.28 to 1.63)	0.07 (−0.02 to 0.16)	1.10 (0.97 to 1.25)
Moderate	0.12 (−0.01 to 0.29)	1.24 (0.98 to 1.54)	−0.02 (−0.18 to 0.15)	0.98 (0.76 to 1.25)
Severe	0.23 (0.02 to 0.44)	1.42 (1.04 to 1.84)	0.08 (−0.14 to 0.31)	1.11 (0.80 to 1.46)
Anxiety disorders				
Mild	0.04 (−0.01 to 0.10)	1.18 (0.98 to 1.39)	0.02 (−0.04 to 0.09)	1.06 (0.86 to 1.35)
Moderate	−0.09 (−0.18 to −0.01)	0.67 (0.31 to 0.95)	−0.11 (−0.22 to −0.01)	0.62 (0.31 to 0.96)
Severe	0.01 (−0.12 to 0.15)	1.03 (0.53 to 1.56)	−0.01 (−0.15 to 0.14)	0.97 (0.48 to 1.52)
Self-harm or suicide^b^				
Mild	0.00 (−0.01 to 0.02)	1.06 (0.56 to 1.71)	−0.00 (−0.02 to 0.02)	0.93 (0.44 to 1.80)
Moderate	0.02 (−0.02 to 0.08)	1.76 (0.38 to 3.71)	0.02 (−0.02 to 0.07)	1.64 (0.43 to 4.18)
Severe	0.00 (−0.04 to 0.06)	1.14 (0.00 to 3.05)	−0.00 (−0.05 to 0.07)	0.94 (0.00 to 3.14)
Any diagnosis of MHC				
Mild	0.50 (0.31 to 0.68)	1.15 (1.09 to 1.21)	0.00 (−0.25 to 0.21)	1.00 (0.93 to 1.06)
Moderate	0.54 (0.17 to 0.91)	1.13 (1.04 to 1.23)	0.05 (−0.36 to 0.48)	1.01 (0.92 to 1.11)
Severe	1.30 (0.74 to 1.83)	1.32 (1.19 to 1.46)	0.79 (0.20 to 1.32)	1.17 (1.04 to 1.30)
Offspring cohort				
Depression				
Mild	0.02 (−0.01 to 0.07)	1.04 (0.97 to 1.13)	−0.02 (−0.07 to 0.04)	0.97 (0.88 to 1.08)
Moderate	0.07 (−0.01 to 0.16)	1.14 (0.98 to 1.33)	0.04 (−0.05 to 0.14)	1.08 (0.91 to 1.26)
Severe	0.06 (−0.05 to 0.17)	1.13 (0.89 to 1.36)	0.03 (−0.10 to 0.15)	1.06 (0.82 to 1.30)
Substance use disorders				
Mild	0.04 (−0.01 to 0.09)	1.08 (0.98 to 1.16)	−0.04 (−0.09 to 0.02)	0.94 (0.86 to 1.03)
Moderate	0.10 (0.00 to 0.20)	1.17 (1.00 to 1.35)	0.03 (−0.08 to 0.14)	1.04 (0.88 to 1.23)
Severe	0.10 (−0.05 to 0.23)	1.18 (0.91 to 1.40)	0.02 (−0.13 to 0.15)	1.03 (0.80 to 1.23)
Anxiety disorders				
Mild	0.03 (0.00 to 0.06)	1.14 (1.02 to 1.31)	0.02 (−0.01 to 0.06)	1.08 (0.94 to 1.30)
Moderate	−0.01 (−0.06 to 0.04)	0.92 (0.69 to 1.23)	−0.02 (−0.07 to 0.04)	0.91 (0.66 to 1.26)
Severe	−0.01 (−0.07 to 0.05)	0.93 (0.60 to 1.30)	−0.01 (−0.08 to 0.06)	0.93 (0.60 to 1.35)
Self-harm or suicide^b^				
Mild	0.02 (0.00 to 0.03)	1.46 (1.12 to 1.84)	0.01 (−0.00 to 0.03)	1.32 (0.89 to 1.87)
Moderate	0.01 (−0.01 to 0.04)	1.37 (0.67 to 2.31)	0.01 (−0.02 to 0.04)	1.23 (0.56 to 2.11)
Severe	0.03 (−0.00 to 0.07)	2.05 (0.85 to 3.35)	0.03 (−0.01 to 0.06)	1.91 (0.82 to 3.23)
Any diagnosis of MHC				
Mild	0.17 (0.09 to 0.25)	1.08 (1.04 to 1.12)	−0.07 (−0.17 to 0.04)	0.97 (0.93 to 1.02)
Moderate	0.10 (−0.07 to 0.25)	1.05 (0.97 to 1.13)	−0.08 (−0.26 to 0.10)	0.96 (0.88 to 1.05)
Severe	0.28 (0.06 to 0.50)	1.15 (1.03 to 1.26)	0.09 (−0.13 to 0.30)	1.04 (0.94 to 1.15)

Abbreviations: GP, general population; MHC, mental health condition; MI, myocardial infarction; RD, risk difference; RR, risk ratio.

^a^ RDs and RRs were weighted for the following variables: age and sex of both study participants and their respective partners or parents, year of index date, household income, highest achieved education, comorbidities (32 distinct conditions), comedications, and health care utilization (full list in eTable 2 in Supplement 1).

^b^ Self-harm or suicide is not included in the composite outcome of any MHCs.

#### Sensitivity Analyses

When depression was defined as either a hospital-based diagnosis or 2 or more prescriptions for an antidepressant with an indication code for depression, the absolute risks markedly increased in all cohorts (eTable 11 in Supplement 1). According to this definition, the RDs and RRs were 1.42% (95% CI, 1.18%-1.59%) and 1.19 (95% CI, 1.1601.21) compared with the GP-partner cohort and 0.41% (95% CI, 0.12%-0.66%) and 1.05 (95% CI, 1.01-1.08) compared with the MI-partner cohort (eTable 12 in Supplement 1). The inclusion of persistent mood disorders in the depression definition had no impact on results (eTables 11 and 12 in Supplement 1). When nonmelanoma skin cancer was used as a negative control outcome, the findings were virtually null (eTables 11 and 12 in Supplement 1). Finally, in a complete case analysis (eTable 13 in Supplement 1) and when the index date was set as the stroke admission date (eTable 14 in Supplement 1), the results were unchanged.

### Adult Children of Stroke Survivors

#### Cohort Characteristics

The offspring cohorts included 207 386 children of stroke survivors (91% ischemic strokes, 68% of mild severity; median [IQR] age, 45 [36-52] years; 99 382 women [48%]), 1 036 886 children of individuals in the GP (median [IQR] age, 45 [36-52] years; 496 875 women [48%]), and 183 309 children of MI survivors (median [IQR] age, 42 [33-49] years; 88 078 women [48%]) (eTable 15 in Supplement 1). As for partners, the baseline characteristics were well balanced even before PS weighting. As expected, the prevalence of comorbidities and drug use was lower in the offspring cohorts than in the partner cohorts (eTable 15 in Supplement 1). After PS weighting, virtually all baseline characteristics had standardized mean differences less than 0.01.

#### Main Analyses

**Figure 2.**
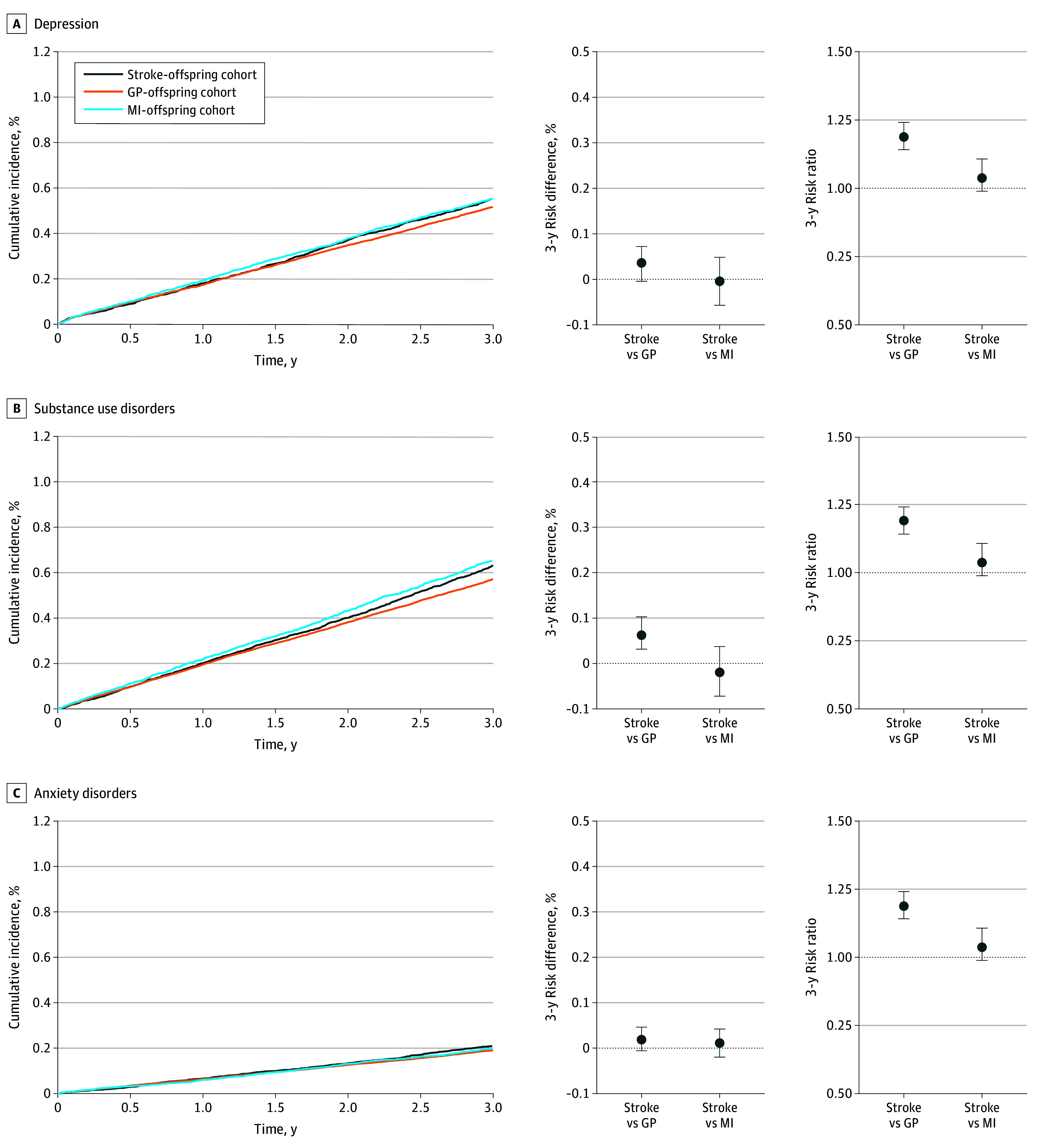
Risk of Mental Health Conditions Among Adult Children of Individuals With Stroke or Myocardial Infarction (MI) and Among the General Population Graphs show propensity score–weighted cumulative incidences, 3-year risk differences, and 3-year risk ratios of depression (A), substance use disorders (B), and anxiety disorders (C) among adult children of stroke survivors (stroke-offspring cohort), adult children of individuals from the general population (GP-offspring cohort), and adult children of MI survivors (MI-offspring cohort). Error bars denote 95% CIs.

Except for self-harm or suicide, the absolute risks for the assessed outcomes were lower in the 3 offspring cohorts than in the 3 partner cohorts. Among children of stroke survivors, the 3-year absolute risk was 0.6% for depression, 0.6% for substance use disorders, 0.2% for anxiety disorders, 0.05% for self-harm or suicide, and 2.2% for the composite outcome (Figure 2 and eTable 4 and eFigure 4 in Supplement 1). RDs and RRs were, in general, closer to the null in these analyses than those reported for the partner analyses (Table 1, Figure 2, and eFigure 4 in Supplement 1). The RRs compared with the GP-offspring cohort were 1.07 (95% CI, 0.99-1.14) for depression, 1.11 (95% CI, 1.05-1.19) for substance use disorders, 1.10 (95% CI, 0.97-1.25) for anxiety disorders, 1.42 (95% CI, 1.11-1.84) for self-harm or suicide, and 1.09 (95% CI, 1.05-1.12) for the composite outcome. Compared with the MI-offspring cohort, RRs were virtually null for all outcomes, except for self-harm or suicide, for which the RR was 1.28 (95% CI, 0.91-1.77).

#### Subgroup Analyses

Stroke severity did not clearly modify the association with depression among adult children (Table 2). Additional subgroup results are presented in eTables 5 to 10 in Supplement 1.

#### Sensitivity Analyses

The results of the sensitivity analyses were in line with those of the sensitivity analyses presented for the partner cohorts. See details in eTables 11 to 14 in Supplement 1.

## Discussion

### Principal Findings

This cohort study examined the 3-year risk of depression, substance use disorders, anxiety disorders, and self-harm or suicide in more than 70 000 partners and more than 200 000 adult children of stroke survivors in Denmark. Our study had 4 main findings: (1) in partners of stroke survivors, RR point estimates for the assessed outcomes ranged from 1.14 to 1.42 compared with the GP and from 1.04 to 1.09 compared with partners of MI survivors; (2) the elevated risk of depression in partners of stroke survivors was more pronounced after severe or moderate stroke than after mild stroke; (3) relative risks found for adult children of stroke survivors were lower than those reported in the partner analyses; and (4) absolute risks were low for all outcomes (eg, 1% and 0.6% for depression in partners and adult children of stroke survivors, respectively). Consequently, despite observed relative risk increases, absolute RDs were marginal in all analyses.

### Comparison With Other Studies

Previous studies used self-administered and interview-based instruments (eg, the Caregiver Strain Index, the Relatives Stress Scale, and the Center for Epidemiologic Studies Depression Scale) to measure the extent of caregiver burden, as well as the presence of symptoms related to depression and anxiety.^14,15,16,17,18,19,20,21,22^ A 2009 systematic review^19^ (24 studies of 2619 caregivers of stroke survivors) found that the prevalence of caregiver burden was 25% to 54% across individual studies. A 2017 meta-analysis^22^ (12 studies of 2059 caregivers of patients with stroke) reported a pooled prevalence of 40% for depressive symptoms and 21% for anxiety symptoms. That meta-analysis^22^ further argued that the estimated pooled prevalence of depression among stroke caregivers is nearly 2-fold higher than the expected prevalence in the GP, citing an older study from 1992.^39^

We believe our results are not directly comparable to those reported previously. Although the instruments used previously are useful in obtaining an assessment of the overall psychological distress associated with stroke caregiving, achieving high scores on such instruments does not in itself meet the criteria for diagnosing a mental health condition. In contrast, our assessment of outcomes relied primarily on diagnoses determined from hospital-based registries.^29,30^ Because mental illness is often unrecognized and, if recognized, is treated in primary care settings with no hospital diagnosis given, registries tend to capture cases of severe mental illness, whereas mild cases are often missed.^40^ For this reason, absolute risks observed in our study were almost certainly underestimated. Indeed, the inclusion of antidepressant use in the definition of depression clearly increased absolute risks; nonetheless, contrasts with either comparison remained largely intact.

Our study population comprised partners and adult children of stroke survivors, some of whom may have served as informal caregivers and others who may not have done so. In contrast, prior studies^14,15,16,17,18,19,20,21,22^ focused exclusively on caregivers. In Denmark, postdischarge stroke rehabilitation is primarily managed by local municipalities.^27^ It is possible this could lead to a comparatively lower caregiver burden for family members, in contrast to countries where patients and their families are often tasked with assuming a more extensive role in the rehabilitation process. Thus, on the basis of the assumption that a component of any true effect of stroke in a family member is mediated by the actual act of caregiving, our estimates may have been diluted toward the null (eFigure 2 in Supplement 1). In support of this possibility, RDs found for adult children, who, on average, may be less involved with informal caregiving than partners, were smaller.

Strokes identified in this study were generally of mild severity (ie, mild stroke comprised 72% of the strokes associated with the stroke-partner cohort and 68% of those associated with the stroke-offspring cohort). The rate of mild stroke has been increasing in Denmark.^3^ This development could be explained by the early establishment of specialized stroke units and an effective prehospital response,^27^ which may have lowered barriers to stroke workup. In contrast, the proportion of mild strokes (according to the National Institute of Health Stroke Scale) was 48% during 2012 to 2015 in the South London Stroke Register (United Kingdom)^41^ and 58% during 1987 to 2009 in the Atherosclerosis Risk in Communities study (US).^42^ Thus, stroke severity is likely to be milder on average in Denmark than in other countries, thereby potentially contributing to the modest overall RDs observed in this study. This possibility was supported by the finding that stroke severity appeared to modify the association between stroke in partners and the risk of depression.

Our study advances existing knowledge by including 2 separate comparison cohorts. As expected, associations were in general larger for the GP comparison than for the MI comparison. Although the effect of MI on long-term disability in survivors tends to be lower than that of stroke,^43^ family members of MI survivors may also experience adverse psychological consequences.^44^ As such, this comparison likely eliminated part of the causal pathway in addition to any potential unmeasured confounding (eFigure 2 in Supplement 1). Notwithstanding, this comparison cohort provided important context to risk estimates, suggesting that stroke and MI convey a roughly similar impact on the psychological consequences in family members.

### Strengths and Limitations

Strengths of this study include its size; its high-quality data within a nationwide, population-based setting with complete long-term follow-up; and its use of comparison cohorts. Positive predictive values of stroke (90%-94%)^45,46^ and MI (97%)^47^ discharge diagnoses are high. Although hospital-based diagnoses of mental health conditions in the Danish registries have positive predictive values of acceptable standards,^30,48,49^ nondifferential misclassification of outcomes may have been present. Motivated by the possibility of unmeasured confounding and diagnostic bias, we included an active comparator cohort, consisting of partners and adult children of MI survivors. Reassuringly, the null result for the negative control outcome (nonmelanoma skin cancer) and the minimal differences in baseline characteristics across cohorts both before and after weighting on a comprehensive set of possible confounders indicate that these sources of potential bias probably did not affect the study results; still, residual confounding is possible.

## Conclusions

Although Denmark has a strong welfare and health care system, where stroke is well managed and stroke severity is relatively mild, no support system exists specifically for family members of stroke survivors, and the high relative risks for self-harm or suicide found in this study for both partners and adult children are concerning. It is possible that upgrading the current organization and services could lessen the burden on family members and lower risks of mental health conditions even more. In summary, this study highlights the potential consequences of stroke among family members, particularly partners, and its findings may possibly serve as a quantitative foundation for the development of future stroke rehabilitation services.

## Notes

Supplement 1. **eAppendix.** Supplemental Methods **eTable 1. **Overview of Studies Assessing Caregiving After Stroke and Various Psychological Aspects of Caregiver Burden **eTable 2.** Codes and Definitions Used in This Study **eTable 3.** Baseline Characteristics (N, %) of Partners of Stroke Patients, Partners of Individuals From the General Population, and Partners of Myocardial Infarction Patients Both Before and After Propensity Score Weighting **eTable 4.** Numbers of Events and 3-Year Absolute Risks of Depression, Substance Use Disorders, Anxiety Disorders, Self-Harm or Suicide, and a Composite Outcome of Any Diagnosis of a Mental Health Condition **eTable 5.** Associations Between Stroke in a Partner or Parent and Risk of Depression, Substance Use Disorders, Anxiety Disorders, Self-Harm or Suicide, and a Composite Outcome of Any Diagnosis of a Mental Health Condition, Stratified by Stroke Subtype **eTable 6.** Associations Between Stroke in a Partner or Parent and Risk of Depression, Substance Use Disorders, Anxiety Disorders, Self-Harm or Suicide, and a Composite Outcome of Any Diagnosis of a Mental Health Condition, Stratified by Age Group **eTable 7.** Associations Between Stroke in a Partner or Parent and Risk of Depression, Substance Use Disorders, Anxiety Disorders, Self-Harm or Suicide, and a Composite Outcome of Any Diagnosis of a Mental Health Condition, Stratified by Sex **eTable 8.** Associations Between Stroke in a Partner or Parent and Risk of Depression, Substance Use Disorders, Anxiety Disorders, Self-Harm or Suicide, and a Composite Outcome of Any Diagnosis of a Mental Health Condition, Stratified by Number of Comorbidities **eTable 9.** Associations Between Stroke in a Partner or Parent and Risk of Depression, Substance Use Disorders, Anxiety Disorders, Self-Harm or Suicide, and a Composite Outcome of Any Diagnosis of a Mental Health Condition, Stratified by Household Income **eTable 10.** Associations Between Stroke in a Partner or Parent and Risk of Depression, Substance Use Disorders, Anxiety Disorders, Self-Harm or Suicide, and a Composite Outcome of Any Diagnosis of a Mental Health Condition, Stratified by Highest Achieved Education **eTable 11.** Numbers of Events and 3-Year Absolute Risks of Depression (Defined From Either a Hospital-Based Diagnosis or ≥2 Prescriptions for an Antidepressant With Indication Code for Depression), Depression (Additionally Including Persistent Mood Disorders), and Nonmelanoma Skin Cancer **eTable 12.** Associations Between Stroke in a Partner or Parent and Risk of Depression (Defined From Either a Hospital-Based Diagnosis or ≥2 Prescriptions for an Antidepressant With Indication Code for Depression), Depression (Additionally Including Persistent Mood Disorders), and Nonmelanoma Skin Cancer **eTable 13.** Associations Between Stroke in a Partner or Parent and Risk of Depression, Substance Use Disorders, Anxiety Disorders, Self-Harm or Suicide, and a Composite Outcome of Any Diagnosis of a Mental Health Condition, When Performing a Complete-Case Analysis in Which Individuals With Missing Data on Household Income or Highest Achieved Education Were Excluded **eTable 14.** Associations Between Stroke in a Partner or Parent and Risk of Depression, Substance Use Disorders, Anxiety Disorders, Self-Harm or Suicide, and a Composite Outcome of Any Diagnosis of a Mental Health Condition, When Performing an Analysis Setting the Index Date to the Stroke Admission Date Instead of the Discharge Date **eTable 15.** Baseline Characteristics (N, %) of Adult Children of Stroke Patients, Adult Children of Individuals From the General Population, and Adult Children of Myocardial Infarction Patients Both Before and After Propensity Score Weighting **eFigure 1.** Flowchart of Study Cohorts **eFigure 2.** Directed Acyclic Graph Depicting Causal Assumptions in This Study **eFigure 3.** Propensity Score Weighted Cumulative Incidences, 3-Year Risk Differences, and 3-Year Risk Ratios of Self-Harm or Suicide and Any Diagnosis of a Mental Health Condition Among Partners of Stroke Survivors (Stroke-Partner Cohort), Partners of Individuals From the General Population (GP-Partner Cohort), and Partners of Myocardial Infarction Survivors (MI-Partner Cohort) **eFigure 4.** Propensity Score Weighted Cumulative Incidences, 3-Year Risk Differences, and 3-Year Risk Ratios of Self-Harm or Suicide and Any Diagnosis of a Mental Health Condition Among Adult Children of Stroke Survivors (Stroke-Offspring Cohort), Adult Children of Individuals From the General Population (GP-Offspring Cohort), and Adult Children of Myocardial Infarction Survivors (MI-Offspring Cohort)
eReferences

Supplement 2.
Data Sharing Statement
