# Supplementary material for: Mental Health Conditions in Partners and Adult Children of Stroke Survivors
Source: JAMA Netw Open. 2024 Mar 14;7(3):e243286. doi: 10.1001/jamanetworkopen.2024.3286 (PMC10940969; doi:10.1001/jamanetworkopen.2024.3286)
Supplement: Supplement 1. — eAppendix. Supplemental Methods eTable 1. Overview of Studies Assessing Caregiving After Stroke and Various Psychological Aspects of Caregiver Burden eTable 2. Codes and Definitions Used in This Study eTable 3. Baseline Characteristics (N, %) of Partners of Stroke Patients, Partners of Individuals From the General Population, and Partners of Myocardial Infarction Patients Both Before and After Propensity Score Weighting eTable 4. Numbers of Events and 3-Year Absolute Risks of Depression, Substance Use Disorders, Anxiety Disorders, Self-Harm or Suicide, and a Composite Outcome of Any Diagnosis of a Mental Health Condition eTable 5. Associations Between Stroke in a Partner or Parent and Risk of Depression, Substance Use Disorders, Anxiety Disorders, Self-Harm or Suicide, and a Composite Outcome of Any Diagnosis of a Mental Health Condition, Stratified by Stroke Subtype eTable 6. Associations Between Stroke in a Partner or Parent and Risk of Depression, Substance Use Disorders, Anxiety Disorders, Self-Harm or Suicide, and a Composite Outcome of Any Diagnosis of a Mental Health Condition, Stratified by Age Group eTable 7. Associations Between Stroke in a Partner or Parent and Risk of Depression, Substance Use Disorders, Anxiety Disorders, Self-Harm or Suicide, and a Composite Outcome of Any Diagnosis of a Mental Health Condition, Stratified by Sex eTable 8. Associations Between Stroke in a Partner or Parent and Risk of Depression, Substance Use Disorders, Anxiety Disorders, Self-Harm or Suicide, and a Composite Outcome of Any Diagnosis of a Mental Health Condition, Stratified by Number of Comorbidities eTable 9. Associations Between Stroke in a Partner or Parent and Risk of Depression, Substance Use Disorders, Anxiety Disorders, Self-Harm or Suicide, and a Composite Outcome of Any Diagnosis of a Mental Health Condition, Stratified by Household Income eTable 10. Associations Between Stroke in a Partner or Parent and Risk of Depression, Substance Use Disorders, Anxiety Di [file jamanetwopen-e243286-s001.pdf]

Supplemental Online Content

Skajaa N, Farkas DK, Laugesen K, et al. Mental health conditions in partners and adult children of stroke survivors. *JAMA Netw Open*. 2024;7(3):e243286.
doi:10.1001/jamanetworkopen.2024.3286

eAppendix. Supplemental Methods
eTable 1. Overview of Studies Assessing Caregiving After Stroke and Various Psychological Aspects of Caregiver Burden
eTable 2. Codes and Definitions Used in This Study
eTable 3. Baseline Characteristics (N, %) of Partners of Stroke Patients, Partners of Individuals From the General Population, and Partners of Myocardial Infarction Patients Both Before and After Propensity Score Weighting
eTable 4. Numbers of Events and 3-Year Absolute Risks of Depression, Substance Use Disorders, Anxiety Disorders, Self-Harm or Suicide, and a Composite Outcome of Any Diagnosis of a Mental Health Condition
eTable 5. Associations Between Stroke in a Partner or Parent and Risk of Depression, Substance Use Disorders, Anxiety Disorders, Self-Harm or Suicide, and a composite Outcome of Any Diagnosis of a Mental Health Condition, Stratified by Stroke Subtype
eTable 6. Associations Between Stroke in a Partner or Parent and Risk of Depression, Substance Use Disorders, Anxiety Disorders, Self-Harm or Suicide, and a Composite Outcome of Any Diagnosis of a Mental Health Condition, Stratified by Age Group
eTable 7. Associations Between Stroke in a Partner or Parent and Risk of Depression, Substance Use Disorders, Anxiety Disorders, Self-Harm or Suicide, and a Composite Outcome of Any Diagnosis of a Mental Health Condition, Stratified by Sex
eTable 8. Associations Between Stroke in a Partner or Parent and Risk of Depression, Substance Use Disorders, Anxiety Disorders, Self-Harm or Suicide, and a Composite Outcome of Any Diagnosis of a Mental Health Condition, Stratified by Number of Comorbidities
eTable 9. Associations Between Stroke in a Partner or Parent and Risk of Depression, Substance Use Disorders, Anxiety Disorders, Self-Harm or Suicide, and a Composite Outcome of Any Diagnosis of a Mental Health Condition, Stratified by Household Income
eTable 10. Associations Between Stroke in a Partner or Parent and Risk of Depression, Substance Use Disorders, Anxiety Disorders, Self-Harm or Suicide, and a Composite Outcome of Any Diagnosis of a Mental Health Condition, Stratified by Highest Achieved Education
eTable 11. Numbers of Events and 3-Year Absolute Risks of Depression (Defined From Either a Hospital-Based Diagnosis or ≥2 Prescriptions for an Antidepressant With Indication Code for Depression), Depression (Additionally Including Persistent Mood Disorders), and Nonmelanoma Skin Cancer
eTable 12. Associations Between Stroke in a Partner or Parent and Risk of Depression (Defined From Either a Hospital-Based Diagnosis or ≥2 Prescriptions for an Antidepressant With Indication Code for Depression), Depression (Additionally Including Persistent Mood Disorders), and Nonmelanoma Skin Cancer
eTable 13. Associations Between Stroke in a Partner or Parent and Risk of Depression, Substance Use Disorders, Anxiety Disorders, Self-Harm or Suicide, and a Composite Outcome of Any Diagnosis of a Mental Health Condition, When Performing a Complete-Case Analysis in

© 2024 Skajaa N et al. *JAMA Network Open.*

Which Individuals With Missing Data on Household Income or Highest Achieved Education Were Excluded
eTable 14. Associations Between Stroke in a Partner or Parent and Risk of Depression, Substance Use Disorders, Anxiety Disorders, Self-Harm or Suicide, and a Composite Outcome of Any Diagnosis of a Mental Health Condition, When Performing an Analysis Setting the Index Date to the Stroke Admission Date Instead of the Discharge Date
eTable 15. Baseline Characteristics (N, %) of Adult Children of Stroke Patients, Adult Children of Individuals From the General Population, and Adult Children of Myocardial Infarction Patients Both Before and After Propensity Score Weighting
eFigure 1. Flowchart of Study Cohorts
eFigure 2. Directed Acyclic Graph Depicting Causal Assumptions in This Study
eFigure 3. Propensity Score Weighted Cumulative Incidences, 3-Year Risk Differences, and 3-Year Risk Ratios of Self-Harm or Suicide and Any Diagnosis of a Mental Health Condition Among Partners of Stroke Survivors (Stroke-Partner Cohort), Partners of Individuals From the General Population (GP-Partner Cohort), and Partners of Myocardial Infarction Survivors (MI-Partner Cohort)
eFigure 4. Propensity Score Weighted Cumulative Incidences, 3-Year Risk Differences, and 3-Year Risk Ratios of Self-Harm or Suicide and Any Diagnosis of a Mental Health Condition Among Adult Children of Stroke Survivors (Stroke-Offspring Cohort), Adult Children of Individuals From the General Population (GP-Offspring Cohort), and Adult Children of Myocardial Infarction Survivors (MI-Offspring Cohort)
eReferences

This supplemental material has been provided by the authors to give readers additional information about their work.

© 2024 Skajaa N et al. *JAMA Network Open.*

eAppendix. Supplemental Methods

Data sources

- The Danish Civil Registration System[1] includes information on date of birth, sex, vital status, and personal identifiers of family members.
- The Danish Stroke Registry[2] collects clinical data on all acute hospital admissions for stroke (*e.g.*, stroke subtype, stroke severity, and in-hospital acute treatment). All hospitals treating patients with stroke, as defined by the World Health Organization criteria, must report to this registry, and diagnoses in this registry have a high positive predictive value (>90%).[3,4]
- The Danish National Patient Registry [5] and the Danish Psychiatric Central Research Registry[6] collectively record all non-psychiatric and psychiatric hospital inpatient and outpatient clinic diagnoses, coded according to the *International Classification of Diseases* (*Eighth Revision* [ICD-8] before 1993 and *Tenth Revision* [ICD-10] thereafter).
- The Danish National Prescription Registry[7] records all prescriptions filled in community pharmacies, coded according to the Anatomical Therapeutic Chemical Classification System.
- The Danish Registry of Causes of Death[8] includes data on dates and causes of deaths, coded according to the ICD-10; information on the cause of death includes the underlying cause of death, the immediate cause of death, contributor causes of death, and additional causes of death.
- Statistics Denmark's income[9] and education[10] registries collectively contain information on personal and household income and the highest level of education completed.

Setting and stroke management

In Denmark, health care is tax-financed and therefore free of personal charge for all residents. Individuals with suspected stroke are transported to and receive treatment in specialized stroke units, comprising a multidisciplinary team of healthcare professionals skilled in both acute care and rehabilitation. The prehospital system pathway and in-hospital organization are operating efficiently: the median time from emergency call to stroke unit arrival (59 minutes) and door-to-needle time (30 minutes for thrombolysis and 121 minutes for thrombectomy) are short.[11] Clinical patient data (*e.g.*, stroke severity according to the Scandinavian Stroke Scale[12] and stroke subtype [*i.e.*, ischemic stroke or intracerebral haemorrhage] as determined from computed tomography or magnetic resonance imaging scans) are mandatorily reported to the Danish Stroke Registry.[2] Specialized stroke rehabilitation is initiated during in-hospital care. However, after hospital discharge, rehabilitation is managed at the municipal level and little data exist regarding its implementation and effectiveness.[13]

© 2024 Skajaa N et al. *JAMA Network Open.*

Study cohorts and follow-up

From the Danish Stroke Registry,[2] we identified patients (≥18 years) hospitalized with a first-time stroke (ischemic stroke or intracerebral hemorrhage) between 1 May 2004 and 31 December 2021 who were discharged from hospital alive. We then assembled two exposed cohorts, *viz.*, partners of stroke survivors (stroke-partner cohort) and adult children of stroke survivors (stroke-offspring cohort) (Supplemental Figure 1). Using linked information in the Civil Registration System,[1] we identified stroke survivors' partners and adult children (linked information on partners and children was available in the registry from 1986; registration of parents was complete for individuals born after 1957). A partnership, on the basis of the most recent information before the stroke diagnosis, was defined according to Statistics Denmark's classification as either (1) two people who were married (including civil unions of same sex couples), (2) two people who were cohabitating with a shared child, or (3) two cohabitating individuals of opposite sex, with an age difference less than 15 years and without a shared child. Regarding children, we considered "legal linkages", in which both biological and adopted children were eligible for inclusion.

We assembled two unexposed comparison cohorts for each of the two exposed cohorts, *viz.*, partners and adult children of individuals from the general population without stroke or MI (GP-partner cohort and GP-offspring cohort) and partners and adult children of myocardial infarction (MI) survivors (MI-partner cohort and MI-offspring cohort). General population comparators were matched 5:1, on birth year and sex, to each member of the stroke-partner and stroke-offspring cohorts using the Civil Registration System. Matching was performed with replacement.[14] Members of the general population who had a partner or parent with a history of stroke or MI were ineligible for matching. We used the Patient Registry[5] to identify patients with a first-time diagnosis of MI between 1 May 2004 and 31 December 2021 who were discharged from hospital alive. Using the same approach as above, we then identified partners and adult children of MI survivors.

In all cohorts, we excluded individuals with a diagnosis of a mental health condition before the index date (defined as the partner's or parent's stroke discharge date for the stroke-partner and stroke-offspring cohorts; the matched partner's or parent's stroke discharge date for the GP-partner and GP-offspring cohorts; and the partner's or parent's MI discharge date for the MI-partner and MI-offspring cohorts) and those <18 years of age (data were not available for those <18 years). Follow-up for all cohort members began on the index date and continued for up to 3 years (data were available until the end of 2021) or until the occurrence of an outcome, death, or emigration, whichever occurred first.

Baseline characteristics

We obtained information on a range of baseline characteristics of the study participants (and their respective partners or parents, when specifically stated). On the basis of the causal assumptions depicted in **eFigure 2**, the following baseline characteristics, measured before the index date, were selected as

© 2024 Skajaa N et al. *JAMA Network Open.*

potential confounders: demographics (*i.e.*, age and sex of both study participants and their respective partners or parents), year of index date, household income, highest achieved education, comorbidity, comedications, and healthcare utilization. Household income was defined as the family's total disposable income after tax, adjusted for family size, and was estimated as the average value using data for the previous 3 years.[15] On the basis of the distribution in the total Danish population according to age and sex, we categorized this variable into low (<25th percentile), medium (25th to <75th percentile), or high (≥75th percentile). We categorized the highest achieved education into low (International Standard Classification of Education [ISCED] level 1–2), medium (ISCED level 3), or high (ISCED level 5–8).[16] To comprehensively measure baseline comorbidity burden, we used a modified version of a previously developed multimorbidity index,[17] which has been extensively used in registry-based studies.[18,19] Our modified version included 32 distinct conditions, which were based on hospital-based diagnoses and, when applicable, redeemed prescriptions (**eTable 2**). We also identified the use of other drugs not used in a definition of an individual comorbidity. Finally, as three indicators of healthcare utilization, we measured the total number of hospital inpatient admissions, outpatient clinic visits, and redeemed prescriptions in the previous 3 years.

In the stroke-partner and stroke-offspring cohorts only, we further obtained information on stroke severity, as measured by the Scandinavian Stroke Scale (mild [43–58], moderate [26–42], or severe [0–25])[12] and stroke subtype (ischemic stroke or intracerebral hemorrhage).

Statistical analyses

We used propensity score (PS) weighting to control for baseline imbalances between cohorts.[20] Using multivariable logistic regression, we predicted the PS for each study participant by conditioning on the baseline patient characteristics measured for all cohorts, as described above. Cohorts were then re-weighted using standardized morbidity ratio weighting, whereby exposed individuals (stroke-partner and stroke-offspring cohorts) were assigned a weight of 1, and unexposed individuals (MI-partner, MI-offspring, GP-partner, and GP-offspring cohorts) were assigned a weight equal to the odds of the exposure probability (PS/1-PS).[20] This weighting approach re-weighted the unexposed cohorts so that their covariate distribution resembled that in the exposed cohorts. In the main analyses, missing data on income (0.2%) and education (1.9%) were handled using a missing data indicator variable in the PS estimation.[21] We assessed covariate balance after weighting using standardized mean differences, with values <0.1 indicative of balance.[22]

We then used the Aalen-Johansen estimator, which accounts for death as a competing event,[23] to calculate weighted 3-year absolute risks, risk differences (RD, difference between absolute risks), and risk ratios (RR, ratio of absolute risks) comparing the stroke-partner/offspring cohorts with the GP-

© 2024 Skajaa N et al. *JAMA Network Open.*

partner/offspring and MI-partner/offspring cohorts. Corresponding 95% confidence intervals (CIs) were obtained through nonparametric bootstrapping (percentile method) using 200 resamples.[24] We also graphically illustrated contrasting risk estimates by constructing weighted cumulative incidence curves.

We repeated the analyses within strata of stroke severity (mild, moderate, or severe), stroke subtype (ischemic stroke or intracerebral hemorrhage), age group (18–54 years, 55–64 years, 65–74 years, or ≥75 years for the partner cohorts, and 18–34 years, 35–44 years, 45–54 years, ≥55 years for the offspring cohorts), sex, number of baseline comorbidities (0, 1, 2, 3, or ≥4), household income (low, medium or high), and highest achieved education (low, medium, or high). In subgroup analyses, PS weights were re-estimated within each examined stratum.[25]

We performed five sensitivity analyses. First, because a hospital-based diagnosis of depression is indicative of severe disease, we redefined depression as either a hospital-based diagnosis or two or more prescriptions for an antidepressant with the indication code for depression, whichever occurred first. By using prescriptions, this alternative outcome definition captured individuals treated in primary care, who to a greater extent may suffer from mild to moderate disease. Second, we additionally included persistent mood disorders (ICD-10: F34) in the depression definition. Third, we used non-melanoma skin cancer as a negative control outcome.[26] Stroke in a partner or parent is expected to be unassociated with non-melanoma skin cancer, particularly given the 3-year exposure window, and potential unmeasured confounding factors, such as smoking,[27] are not clearly associated with non-melanoma skin cancer. Thus, an observed positive association with this outcome could reveal the presence of diagnostic bias, arising from a greater referral rate from primary care to hospital care for partners and adult children of stroke survivors than for comparators. In this analysis, cohort members with a diagnosis of non-melanoma skin cancer before the index date were excluded. Fourth, we performed a complete case analysis, in which individuals with missing values for household income or highest achieved education were excluded. Fifth, we redefined the study population by setting the index date as the stroke admission date instead of the discharge date.

© 2024 Skajaa N et al. *JAMA Network Open.*

eTable 1. Overview of Studies Assessing Caregiving After Stroke and Various Psychological Aspects of Caregiver Burden

Author, journal, year	Design, setting, period	Study population (composition, size)	Outcome	Main findings
**Draper et al**[28]<br>J Am Geriatr Soc<br>1992	Cross-sectional<br>Community rehabilitation and geriatric service (U.K.)<br>Period unknown	Co-resident caregivers of stroke and dementia survivors<br>n = 99	Caregiver burden ("Relative Stress Scale", "General Health Questionnaire")	46% had significant psychological morbidity
**Anderson et al**[29]<br>Stroke<br>1995	Cross-sectional<br>Perth Community Stroke Study (Australia)<br>1989-1990	Caregivers of one-year stroke survivors<br>n = 84	Caregiver burden ("Hospital Anxiety and Depression Scale", "General Health Questionnaire")	55% had emotional distress. Almost reported adverse effects on emotional health, social activities, and leisure time
**Berg et al**[30]<br>Stroke<br>2005	Cohort study<br>Hospital-based (Finland)<br>Period unknown	Caregivers of ischemic stroke survivors<br>n = 98	Depression ("Beck Depression Inventory"<br>Measurements done at baseline, 6 months, and 18 months after stroke	Depression prevalence during follow-up: 30%-33%
**Draper et al**[31]<br>J Clin Nurs<br>2007	Cross-sectional<br>Hospital-based (U.K.)<br>Period unknown	Stroke survivors (within 12 months) and their partner<br>n = 44	Caregiver burden ("General Health Questionnaire" and "Caregiver Strain Index")	A large proportion found that caregiving was stressful, stroke disability was not directly related to extent of psychiatric morbidity and strain in spouses
**Rigby et al**[32]<br>Int J Stroke<br>2009	Systematic review<br>Studies reporting on caregiver burden following stroke<br>Studies published before 2008	Caregivers of stroke survivors<br>n = 24 studies (total sample size = 2,619)	Nine different instruments to assess caregiver burden	Prevalence of caregiver burden ranged from 25% to 54% across studies
**Rigby et al**[33]<br>Int J Stroke<br>2009	Cohort study<br>Hospital-based (Canada)<br>2011-2002	Caregivers of stroke survivors<br>n = 155	Caregiver burden ("Relative Stress Scale, "Bakas Caregiver Outcomes Scale")<br>Measurements done at 12 months after stroke	Impaired functional status at baseline (patient) was associated with caregiver burden at 12 months
**Cameron et al**[34]<br>Stroke<br>2011	Cohort study<br>Hospital-based (Canada)<br>Period unknown	Caregivers of stroke survivors<br>n = 399	Behavioral and psychological symptoms ("Brain Impairment Behavior Inventory-Revised"), caregiver emotional distress ("Center for Epidemiological Studies Depression Scale")<br>Measurements done at 1, 3, 6, 12, 18, and 24 months after stroke	Caregiver emotional distress was associated with depressive symptoms and cognitive decline in the stroke survivor
**Haley et al**[35]<br>Neurology<br>2015	Cohort study<br>Reasons for Geographic and Racial Differences in Stroke (U.S.)	Partners acting as caregivers to stroke survivors (n = 235) and matched non-caregivers (n = 235)	Emotional distress ("Center for Epidemiological Studies Depression Scale"), quality of	Caregivers had poorer well-being than controls at 9 months on almost all

© 2024 Skajaa N et al. *JAMA Network Open.*

	2003-2007		life ("Short From Health Survey"), life satisfaction ("Life Satisfaction Index-Z), leisure activity satisfaction ("Leisure Time Satisfaction" scale)<br>Measurements done at 9, 18, 27, and 36 months after stroke	instrument; difference decreased from 9 months until 36 months
**Loh et al**[36]<br>J Am Med Dir Assoc<br>2017	Meta-analysis<br>Studies reporting on prevalence of anxiety and depressive symptoms among caregivers of stroke survivors<br>Studies published before 2016	Caregivers of stroke survivors<br>n = 12 studies (total sample size = 2,059)	Anxiety and depression as based on standardized instruments or interviews	Prevalence of anxiety symptoms = 21%; prevalence of depressive symptoms = 40%

© 2024 Skajaa N et al. *JAMA Network Open.*

eTable 2. Codes and Definitions Used in This Study

	Code or variable	Definition	Registry used
**Study population**			
Ischemic stroke	Apotype: 3, 4 (ICD-10 equivalent codes: I63, I64)	Record indicating hospitalization with acute stroke	DSR
Intracerebral hemorrhage	Apotype: 2 (ICD-10 equivalent codes: I61)	Record indicating hospitalization with acute stroke	DSR
Myocardial infarction	ICD-10: I21	Diagnosis (any type)	DNPR
**Exclusion criteria**			
Mental health conditions	ICD-10 codes: F00-F99, G30, X60-X84<br>ICD-8 codes: 290.09, 290.10, 290.11, 290.18, 290.19, 292.x9, 293.x9, 294.x9, 309.x9, 291.x9, 294.39, 303.x9, 303.20, 303.28, 303.90, 304.x9, 295.x9, 296.89, 297.x9, 298.29-298.99, 299.04, 299.05, 299.09, 301.83, 296.x9 (excl. 296.89), 298.09, 298.19, 300.49, 301.19, 300.x9 (excl. 300.49), 305.x9, 305.68, 307.99, 305.60, 306.50, 306.58, 306.59, 301.x9 (excl. 301.19), 301.80, 301.81, 301.82, 301.84, 311.xx, 312.xx, 313.xx, 314.xx, 315.xx, 299.00, 299.01, 299.02, 299.03, 306.x9, 308.0x)	Diagnosis (any type) before the index date	DNPR, DPCRR
Stroke	Apotype: 2, 3, 4<br>ICD-10 codes: I60, I61, I63, I64<br>ICD-8 codes: 430, 431, 433, 434	Record indicating hospitalization with acute stroke OR diagnosis (any type) before the index date	DSR, DNPR
Myocardial infarction	ICD-10 codes: I21<br>ICD-8 codes: 410	Diagnosis (any type) before the index date	DNPR
**Patient characteristics**			
Demographics			
Household income	AEKVIVADISP_13	Income groups was calculated based on a 3-year average in the total population	IND
Highest achieved education	HFAUDD	Education groups was calculated based on ISCED levels. The most recent data, going back three years, were used	PER
Comorbidities			
Hypertension	ICD-10 codes: I10-I13, I15<br>ATC codes: C02-C04, C07-C09	Diagnosis (any type) before the index date OR ≥2 prescriptions in last year before the index date; however, if also ischemic heart disease or heart failure, don't include based on prescriptions; if also chronic kidney disease, don't include based on diuretics (C03)	DNPR, NPR
Dyslipidemia	ICD-10 codes: E78<br>ATC codes: C10	Diagnosis (any type) in last two years before the index date OR ≥2 prescriptions in last year before the index date	DNPR, NPR
Ischemic heart disease	ICD-10 codes:I20-I25<br>ATC codes:C01DA	Diagnosis (any type) before the index date OR ≥2 prescriptions in last year before the index date	DNPR, NPR
Atrial fibrillation	ICD-10 codes: I48	Diagnosis (any type) before the index date	DNPR
Heart failure	ICD-10 codes: I50	Diagnosis (any type) before the index date	DNPR
Peripheral artery disease	ICD-10 codes: I70-I74	Diagnosis (any type) before the index date	DNPR
Venous thromboembolism	ICD-10 codes: I801-I803, I26	Diagnosis (any type) before the index date	DNPR
Stroke	ICD-10 codes: I60-I61, I63-I64	Diagnosis (any type) before the index date	DNPR
Diabetes mellitus	ICD-10 codes: E10-E14<br>ATC codes: A10A, A10B	Diagnosis (any type) before the index date OR ≥2 prescriptions in last year before the index date	DNPR, NPR
Thyroid disorder	ICD-10 codes: E00-E05, E061-E069, E07<br>ATC codes: H03	Diagnosis (any type) in last two years before the index date OR ≥2 prescriptions in last year before the index date	DNPR, NPR
Gout	ICD-10 codes: E79, M10	Diagnosis (any type) before the index date	DNPR
Obstructive pulmonary disease	ICD-10 codes: J40-J46<br>ATC codes: R03	Diagnosis (any type) before the index date OR ≥2 prescriptions in last year before the index date	DNPR, NPR
Allergy	ATC codes: R06AX, R06AE07, R06AE09, R01AC, R01AD	≥2 prescriptions in last year before the index date	NPR
Ulcer/chronic gastritis	ICD-10 codes: K211, K25-K28, K293-K295	Diagnosis (any type) before the index date	DNPR
Chronic liver disease	ICD-10 codes: B16-B19, K70-K74, K766, I85	Diagnosis (any type) before the index date	DNPR
Inflammatory bowel disease	ICD-10 codes: K50-K51	Diagnosis (any type) before the index date	DNPR

© 2024 Skajaa N et al. *JAMA Network Open.*

Diverticular disease	ICD-10 codes: K57	Diagnosis (any type) before the index date	DNPR
Chronic kidney disease	ICD-10 codes: N03, N11, N18-N19	Diagnosis (any type) before the index date	DNPR
Prostate disorders	ICD-10 codes: N40<br>ATC codes: C02CA, G04C	Diagnosis (any type) before the index date OR ≥2 prescriptions in last year before the index date	DNPR, NPR
Connective tissue disorders	ICD-10 codes: M05-M06, M08-M09, M30-M36, D86	Diagnosis (any type) before the index date	DNPR
Osteoporosis	ICD-10 codes: M80-M82, S120-S122, S127, S129, S220-S221, T08, S32, S422-S424, S427-S428, S52, S62, S720-S722<br>ATC codes: M05B, G03XC01, H05AA	Diagnosis (any type) before the index date OR ≥2 prescriptions in last year before the index date	DNPR, NPR
Painful condition	ATC codes: N02A, N02BA51, N02BE	Diagnosis (any type) before the index date OR ≥4 prescriptions in last year before the index date	DNPR, NPR
HIV/AIDS	ICD-10 codes: B20-B24	Diagnosis (any type) before the index date	DNPR
Anemias	ICD-10 codes: D50-D53, D55-59, D60-D61, D63-D64	Diagnosis (any type) in last two years before the index date	DNPR
Cancer	ICD-10 codes: C00-C99 (excl. C44)	Diagnosis (any type) in last five years before the index date	
Vision problem	ICD-10 codes: N40, N25, H54	Diagnosis (any type) before the index date	DNPR
Hearing	ICD-10 codes: H90-H91, H931	Diagnosis (any type) before the index date	DNPR
Migraine	ICD-10 codes: G43<br>ATC codes: N02C	Diagnosis (any type) in last two years the index date OR ≥2 prescriptions in last year before the index date	DNPR, NPR
Epilepsy	ICD-10 codes: G40-G47<br>ATC codes: N03	Diagnosis (any type) before the index date OR ≥2 prescriptions in last year before the index date	DNPR, NPR
Parkinson's disease	ICD-10 codes: G20-G22	Diagnosis (any type) before the index date	DNPR
Multiple sclerosis	ICD-10 codes: G35	Diagnosis (any type) before the index date	DNPR
Neuropathies	ICD-10 codes: G50-G64	Diagnosis (any type) in last two years before the index date	DNPR
Other medications			
Antidepressants	ATC codes: N06A	≥1 prescriptions in last year before the index date	NPR
Anti-dementia drugs	ATC codes: N06D	≥1 prescriptions in last year before the index date	NPR
Drugs used in additive disorders	ATC codes: N07BB, N07BC	≥1 prescriptions in last year before the index date	NPR
Antipsychotics	ATC codes: N05A	≥1 prescriptions in last year before the index date	NPR
Anxiolytics	ATC codes: N05B	≥1 prescriptions in last year before the index date	NPR
Hypnotics/sedatives	ATC codes: N05C	≥1 prescriptions in last year before the index date	NPR
Antiplatelets	ATC codes: B01AC06, B01AC30, B01AC07, B01AC04, B01AC22, B01AC24	≥1 prescriptions in last year before the index date	NPR
Anticoagulants	ATC codes: B01AA, B01AE07, B01AF01, B01AF02, B01AF03	≥1 prescriptions in last year before the index date	NPR
NSAIDs	ATC codes: M01AA-M01AH, M01AX01	≥1 prescriptions in last year before the index date	NPR
Systemic glucocorticoids	ATC codes: H02AB01, H02AB04, H02AB06-H02AB09	≥1 prescriptions in last year before the index date	NPR
Proton pump inhibitors	ATC codes: A02BC	≥1 prescriptions in last year before the index date	NPR
**Outcomes**			
Depression	ICD-10 codes: F32-F33	Diagnosis (inpatient/outpatient, primary/secondary) after or on the index date	DNPR, DPCRR
Substance use disorders	ICD-10 codes: F10-F19	Diagnosis (inpatient/outpatient, primary/secondary) after or on the index date	DNPR, DPCRR
Anxiety disorders	ICD-10 codes: F41	Diagnosis (inpatient/outpatient, primary/secondary) after or on the index date	DNPR, DPCRR
Self-harm or suicide	ICD-10 codes: X60-X84	Diagnosis (inpatient/outpatient, primary/secondary) OR cause of death (immediate, underlying) after or on the index date.	DNPR, DPCRR, DRCD
Any mental health condition	ICD-10 codes: F00-F99, G30	Diagnosis (inpatient/outpatient, primary/secondary) after or on the index date	DNPR, DPCRR
**Sensitivity analyses**			
Depression	ICD-10 codes: F32-F33<br>ATC codes: N06A	Diagnosis (inpatient/outpatient, primary/secondary) OR ≥2 prescriptions after or on the index date with the indication code 0000168 ("for depression") after or on the index date	DNPR, DPCRR, NPR
Depression	ICD-10 codes: F32-F34	Diagnosis (inpatient/outpatient, primary/secondary) after or on the index date	DNPR, DPCRR
Non-melanoma skin cancer	ICD-10 codes: C44	Diagnosis (inpatient/outpatient, primary/secondary) after or on the index date	DNPR

© 2024 Skajaa N et al. *JAMA Network Open.*

Abbreviations: ICD: International Classification of Disease; ATC: Anatomical Therapeutic Chemical; DSR: Danish Stroke Registry; DNPR: Danish National Patient Registry; DPCRR: Danish Psychiatric Central Research Database; IND: The Income Statistics Registry; PER: Population Education Registry; NPR: Danish National Prescription Registry; DRCD: Danish Registry of Causes of Death; ISCED: International Standard Classification of Education; NSAID: non-steroidal anti-inflammatory drugs.

© 2024 Skajaa N et al. *JAMA Network Open.*

eTable 3. Baseline Characteristics (N, %) of Partners of Stroke Patients, Partners of Individuals From the General Population, and Partners of Myocardial Infarction Patients Both Before and After Propensity Score Weighting

	Unweighted cohorts			PS-weighted cohorts			
	**Stroke-partner cohort**	**GP-partner cohort**	**MI-partner cohort**	**GP-partner cohort**	**SMD**	**MI-partner cohort**	**SMD**
**Overall**	70917	354570	70664	70875	-	70951	-
**Age, years (median, IQR)**	68 (59, 76)	68 (59, 76)	65 (55, 73)	68 (59, 76)	0.00	68 (59, 76)	0.00
**Age of partner, years (median, IQR)**	70 (61, 77)	68 (59, 76)	66 (57, 75)	70 (61, 77)	0.00	69 (61, 77)	0.00
**Sex, n (%)**							
Women	46369 (65)	231833 (65)	51849 (73)	46391 (65)	0.01	46122 (65)	0.00
Men	24548 (35)	122737 (35)	18815 (27)	24483 (35)	-0.01	24830 (35)	0.00
**Sex of partner, n (%)**							
Women	24493 (35)	122981 (35)	18742 (27)	24437 (34)	-0.01	24774 (35)	0.00
Men	46424 (65)	231589 (65)	51922 (73)	46438 (66)	0.01	46177 (65)	0.00
**Household income, n (%)**							
Low (<25th percentile)	23140 (33)	109216 (31)	24039 (34)	23152 (33)	0.00	23161 (33)	0.00
Medium (25th-<75th percentile)	32779 (46)	160492 (45)	33297 (47)	32726 (46)	0.00	32812 (46)	0.00
High (≥75th percentile)	14980 (21)	84801 (24)	13298 (19)	14979 (21)	0.00	14960 (21)	0.00
**Highest achieved education, n (%)**							
Low (ISCED level 1-2)	25931 (37)	114754 (32)	25559 (36)	25973 (37)	0.00	26021 (37)	0.00
Medium (ISCED level 3)	27878 (39)	138411 (39)	28019 (40)	27842 (39)	0.00	27932 (39)	0.00
High (ISCED level 5-8)	14495 (20)	88977 (25)	14167 (20)	14467 (20)	0.00	14389 (20)	0.00
**Comorbidity, n (%)**							
Hypertension	28750 (41)	139513 (39)	25642 (36)	28802 (41)	0.00	28796 (41)	0.00
Dyslipidemia	16659 (23)	82170 (23)	14389 (20)	16690 (24)	0.00	16697 (24)	0.00
Ischemic heart disease	7815 (11)	36182 (10)	6830 (10)	7848 (11)	0.00	7834 (11)	0.00
Atrial fibrillation	4392 (6)	21415 (6)	3481 (5)	4405 (6)	0.00	4411 (6)	0.00
Heart failure	2080 (3)	9276 (3)	1678 (2)	2085 (3)	0.00	2080 (3)	0.00
Peripheral artery disease	2670 (4)	11685 (3)	2234 (3)	2679 (4)	0.00	2670 (4)	0.00
Venous thromboembolism	1608 (2)	7775 (2)	1452 (2)	1612 (2)	0.00	1604 (2)	0.00
Stroke	3237 (5)	14960 (4)	2678 (4)	3250 (5)	0.00	3266 (5)	0.00
Diabetes mellitus	6194 (9)	27126 (8)	5558 (8)	6206 (9)	0.00	6221 (9)	0.00
Thyroid disorders	4139 (6)	20982 (6)	4244 (6)	4142 (6)	0.00	4124 (6)	0.00
Gout	634 (1)	2600 (1)	485 (1)	636 (1)	0.00	638 (1)	0.00
Obstructive pulmonary disease	7686 (11)	35892 (10)	7392 (10)	7699 (11)	0.00	7723 (11)	0.00
Ulcer/chronic gastritis	2096 (3)	9929 (3)	1910 (3)	2101 (3)	0.00	2097 (3)	0.00
Chronic liver disease	435 (1)	1804 (1)	391 (1)	436 (1)	0.00	431 (1)	0.00
Inflammatory bowel disease	741 (1)	3969 (1)	806 (1)	740 (1)	0.00	741 (1)	0.00
Diverticular disease	2986 (4)	14388 (4)	2481 (4)	2988 (4)	0.00	2993 (4)	0.00
Chronic kidney disease	759 (1)	3577 (1)	635 (1)	761 (1)	0.00	766 (1)	0.00
Prostate disorders	4046 (6)	20880 (6)	3025 (4)	4045 (6)	0.00	4037 (6)	0.00
Connective tissue disorders	2389 (3)	12258 (3)	2322 (3)	2394 (3)	0.00	2387 (3)	0.00
Osteoporosis	13744 (19)	68050 (19)	12516 (18)	13780 (19)	0.00	13803 (19)	0.00
HIV/AIDS	31 (0)	89 (0)	38 (0)	28 (0)	0.00	31 (0)	0.00

© 2024 Skajaa N et al. *JAMA Network Open.*

Anemias	714 (1)	3224 (1)	662 (1)	712 (1)	0.00	724 (1)	0.00
Cancer	4596 (6)	23349 (7)	4105 (6)	4602 (6)	0.00	4586 (6)	0.00
Vision problems	2714 (4)	13856 (4)	2078 (3)	2715 (4)	0.00	2707 (4)	0.00
Hearing problems	7251 (10)	37031 (10)	6067 (9)	7271 (10)	0.00	7292 (10)	0.00
Migraine	1011 (1)	5059 (1)	1215 (2)	1010 (1)	0.00	1004 (1)	0.00
Epilepsy	5606 (8)	26661 (8)	5188 (7)	5612 (8)	0.00	5617 (8)	0.00
Parkinson’s disease	223 (0)	1145 (0)	190 (0)	225 (0)	0.00	221 (0)	0.00
Multiple sclerosis	223 (0)	1081 (0)	263 (0)	221 (0)	0.00	218 (0)	0.00
Neuropathies	1003 (1)	4364 (1)	924 (1)	1002 (1)	0.00	993 (1)	0.00
Allergy	3622 (5)	18907 (5)	3613 (5)	3627 (5)	0.00	3633 (5)	0.00
Painful condition	7205 (10)	32616 (9)	6883 (10)	7240 (10)	0.00	7186 (10)	0.00
**Use of medications, n (%)**							
Antidepressants	5616 (8)	26165 (7)	5758 (8)	5634 (8)	0.00	5594 (8)	0.00
Anti-dementia drugs	106 (0)	497 (0)	64 (0)	107 (0)	0.00	107 (0)	0.00
Drugs used in addictive disorders	247 (0)	919 (0)	199 (0)	244 (0)	0.00	252 (0)	0.00
Antipsychotics	663 (1)	2982 (1)	670 (1)	667 (1)	0.00	663 (1)	0.00
Anxiolytics	3916 (6)	16796 (5)	3849 (5)	3938 (6)	0.00	3934 (6)	0.00
Hypnotics and sedatives	6247 (9)	28189 (8)	5501 (8)	6269 (9)	0.00	6266 (9)	0.00
Antiplatelets	13538 (19)	63983 (18)	11599 (16)	13579 (19)	0.00	13585 (19)	0.00
Anticoagulants	4152 (6)	20086 (6)	3168 (4)	4160 (6)	0.00	4155 (6)	0.00
NSAIDs	13826 (19)	66390 (19)	14869 (21)	13817 (19)	0.00	13828 (19)	0.00
Systemic glucocorticoids	4016 (6)	19480 (5)	3856 (5)	4028 (6)	0.00	4038 (6)	0.00
Proton pump inhibitors	11177 (16)	53484 (15)	10638 (15)	11209 (16)	0.00	11204 (16)	0.00
**Number of inpatient admissions in previous 3 years, n (%)**							
0	49004 (69)	249557 (70)	49566 (70)	48955 (69)	0.00	49001 (69)	0.00
1	12290 (17)	60228 (17)	12056 (17)	12277 (17)	0.00	12298 (17)	0.00
≥2	9623 (14)	44785 (13)	9042 (13)	9643 (14)	0.00	9652 (14)	0.00
**Number of outpatient visits in previous 3 years, n (%)**							
0	20544 (29)	100375 (28)	20858 (30)	20508 (29)	0.00	20522 (29)	0.00
1-3	20273 (29)	103436 (29)	20842 (29)	20256 (29)	0.00	20305 (29)	0.00
4-7	12875 (18)	64599 (18)	12776 (18)	12869 (18)	0.00	12872 (18)	0.00
≥8	17225 (24)	86160 (24)	16188 (23)	17242 (24)	0.00	17252 (24)	0.00
**Number of redeemed prescriptions in previous 3 years, n (%)**							
0-5	17220 (24)	89136 (25)	18397 (26)	17137 (24)	0.00	17218 (24)	0.00
6-15	11701 (16)	60159 (17)	12854 (18)	11680 (16)	0.00	11729 (17)	0.00
16-35	15210 (21)	77728 (22)	15211 (22)	15216 (21)	0.00	15183 (21)	0.00
≥36	26786 (38)	127547 (36)	24202 (34)	26841 (38)	0.00	26821 (38)	0.00
**Stroke severity according to the Scandinavian Stroke Scale, n (%)**							
Mild (SSS score 43-58)	50707 (72)	-	-	-	-	-	-
Moderate (SSS score 26-42)	9822 (14)	-	-	-	-	-	-
Severe or very severe (SSS score 0-25)	5872 (8)	-	-	-	-	-	-

© 2024 Skajaa N et al. *JAMA Network Open.*

Missing	4516 (6)	-	-	-	-	-	-
**Stroke subtype, n (%)**							
Ischemic stroke	64338 (91)	-	-	-	-	-	-
Intracerebral hemorrhage	6579 (9)	-	-	-	-	-	-

Abbreviations: GP: general population; MI: myocardial infarction; PS: propensity score; SMD: standardized mean difference; IQR: interquartile range; ISCED: International Standard Classification of Education; NSAID: non-steroidal anti-inflammatory drugs; SSS: Scandinavian Stroke Scale.

© 2024 Skajaa N et al. *JAMA Network Open.*

eTable 4. Numbers of Events and 3-Year Absolute Risks of Depression, Substance Use Disorders, Anxiety Disorders, Self-Harm or Suicide, and a Composite Outcome of Any Diagnosis of a Mental Health Condition

	Events, N [a]	3-year absolute risk, % [a]
**Partner cohort**		
Depression		
Stroke	666	1.03
GP	2359	0.74
MI	600	0.91
Substance use disorders		
Stroke	475	0.73
GP	1535	0.48
MI	437	0.67
Anxiety disorders		
Stroke	185	0.29
GP	754	0.24
MI	189	0.29
Self-harm or suicide [b]		
Stroke	24	0.04
GP	89	0.03
MI	22	0.03
Any diagnosis of a mental health condition		
Stroke	2624	4.05
GP	10589	3.29
MI	2314	3.53
**Offspring cohort**		
Depression		
Stroke	1040	0.55
GP	4624	0.49
MI	1025	0.60
Substance use disorders		
Stroke	1183	0.63
GP	5157	0.55
MI	1183	0.70
Anxiety disorders		
Stroke	394	0.21
GP	1719	0.18
MI	363	0.21
Self-harm or suicide [b]		
Stroke	86	0.05
GP	286	0.03
MI	70	0.04
Any diagnosis of a mental health condition		
Stroke	4127	2.19
GP	18299	1.94
MI	4133	2.43

Abbreviations: CI: confidence interval; GP: general population; MI: myocardial infarction.
[a] Numbers of events and absolute risks were calculated in the original, unweighted population.
[b] Self-harm or suicide was not included in the composite outcome of any mental health conditions.

© 2024 Skajaa N et al. *JAMA Network Open.*

eTable 5. Associations Between Stroke in a Partner or Parent and Risk of Depression, Substance Use Disorders, Anxiety Disorders, Self-Harm or Suicide, and a composite Outcome of Any Diagnosis of a Mental Health Condition, Stratified by Stroke Subtype

		Stroke *vs.* GP		Stroke *vs.* MI	
		**3-year risk difference, % (95% CI) [a]**	**3-year risk ratio (95% CI) [a]**	**3-year risk difference, % (95% CI) [a]**	**3-year risk ratio (95% CI) [a]**
**Partner cohort**					
Depression	Ischemic stroke	0.26 (0.17, 0.34)	1.33 (1.21, 1.44)	0.10 (-0.02, 0.20)	1.10 (0.98, 1.21)
	Intracerebral hemorrhage	0.08 (-0.12, 0.33)	1.10 (0.85, 1.43)	-0.10 (-0.35, 0.16)	0.90 (0.66, 1.17)
Substance use disorders	Ischemic stroke	0.24 (0.16, 0.31)	1.46 (1.31, 1.61)	0.08 (-0.03, 0.19)	1.12 (0.96, 1.30)
	Intracerebral hemorrhage	0.05 (-0.15, 0.26)	1.10 (0.70, 1.53)	-0.10 (-0.31, 0.10)	0.85 (0.54, 1.15)
Anxiety disorders	Ischemic stroke	0.04 (-0.01, 0.08)	1.15 (0.97, 1.33)	0.01 (-0.05, 0.07)	1.04 (0.86, 1.29)
	Intracerebral hemorrhage	0.02 (-0.12, 0.14)	1.06 (0.54, 1.53)	0.00 (-0.15, 0.13)	0.98 (0.49, 1.47)
Self-harm or suicide [b]	Ischemic stroke	0.01 (-0.01, 0.03)	1.38 (0.84, 2.03)	0.01 (-0.01, 0.03)	1.20 (0.68, 2.44)
	Intracerebral hemorrhage	-0.03 (-0.04, -0.02)	0.00 (0.00, 0.00)	-0.03 (-0.05, -0.02)	0.00 (0.00, 0.00)
Any diagnosis of a MHC	Ischemic stroke	0.66 (0.49, 0.83)	1.19 (1.14, 1.24)	0.16 (-0.05, 0.38)	1.04 (0.99, 1.10)
	Intracerebral hemorrhage	0.50 (0.05, 0.99)	1.15 (1.01, 1.29)	-0.02 (-0.48, 0.50)	1.00 (0.88, 1.13)
**Offspring cohort**					
Depression	Ischemic stroke	0.03 (-0.01, 0.07)	1.06 (0.98, 1.14)	-0.01 (-0.06, 0.04)	0.98 (0.90, 1.08)
	Intracerebral hemorrhage	0.10 (-0.01, 0.24)	1.21 (0.98, 1.48)	0.07 (-0.05, 0.20)	1.12 (0.91, 1.37)
Substance use disorders	Ischemic stroke	0.06 (0.02, 0.11)	1.10 (1.03, 1.19)	-0.02 (-0.07, 0.03)	0.97 (0.90, 1.05)
	Intracerebral hemorrhage	0.10 (-0.01, 0.19)	1.18 (0.99, 1.36)	0.01 (-0.09, 0.13)	1.02 (0.86, 1.22)
Anxiety disorders	Ischemic stroke	0.02 (-0.01, 0.04)	1.08 (0.95, 1.21)	0.01 (-0.02, 0.04)	1.04 (0.91, 1.20)
	Intracerebral hemorrhage	0.05 (-0.03, 0.12)	1.27 (0.85, 1.65)	0.04 (-0.04, 0.12)	1.19 (0.82, 1.61)
Self-harm or suicide [b]	Ischemic stroke	0.01 (0.00, 0.03)	1.44 (1.09, 1.84)	0.01 (0.00, 0.02)	1.30 (0.92, 1.78)
	Intracerebral hemorrhage	0.01 (-0.02, 0.03)	1.17 (0.23, 1.98)	0.00 (-0.03, 0.03)	1.09 (0.23, 1.98)
Any diagnosis of a MHC	Ischemic stroke	0.15 (0.10, 0.22)	1.08 (1.05, 1.11)	-0.07 (-0.16, 0.01)	0.97 (0.93, 1.00)
	Intracerebral hemorrhage	0.39 (0.18, 0.61)	1.20 (1.09, 1.31)	0.17 (-0.07, 0.38)	1.08 (0.97, 1.18)

Abbreviations: CI: confidence interval; GP: general population; MI: myocardial infarction; MHC: mental health condition.

[a] Risk differences and risk ratios were weighted for the following variables: age and sex of both study participants and their respective partners or parents, year of index date, household income, highest achieved education, comorbidities (32 distinct conditions), comedications, and healthcare utilization (full list in **eTable 2**).

[b] Self-harm or suicide was not included in the composite outcome of any mental health conditions.

© 2024 Skajaa N et al. *JAMA Network Open.*

eTable 6. Associations Between Stroke in a Partner or Parent and Risk of Depression, Substance Use Disorders, Anxiety Disorders, Self-Harm or Suicide, and a Composite Outcome of Any Diagnosis of a Mental Health Condition, Stratified by Age Group

		**Stroke *vs.* GP**		**Stroke *vs.* MI**	
		**3-year risk difference, % (95% CI)** [a]	**3-year risk ratio (95% CI)** [a]	**3-year risk difference, % (95% CI)** [a]	**3-year risk ratio (95% CI)** [a]
**Partner cohort**					
Depression	Age <55 years	0.19 (0.02, 0.38)	1.36 (1.03, 1.75)	-0.04 (-0.23, 0.18)	0.94 (0.73, 1.25)
	Age 55-64 years	0.18 (0.03, 0.32)	1.47 (1.06, 1.86)	0.07 (-0.11, 0.24)	1.15 (0.81, 1.58)
	Age 65-74 years	0.21 (0.08, 0.34)	1.33 (1.13, 1.59)	0.17 (0.01, 0.33)	1.25 (1.02, 1.60)
	Age ≥75 years	0.39 (0.20, 0.59)	1.26 (1.13, 1.41)	0.09 (-0.18, 0.35)	1.05 (0.90, 1.22)
Substance use disorders	Age <55 years	0.21 (0.04, 0.37)	1.43 (1.07, 1.87)	-0.13 (-0.34, 0.07)	0.84 (0.62, 1.10)
	Age 55-64 years	0.33 (0.19, 0.50)	1.57 (1.31, 1.89)	0.27 (0.05, 0.45)	1.41 (1.06, 1.80)
	Age 65-74 years	0.20 (0.06, 0.34)	1.34 (1.10, 1.60)	0.14 (-0.04, 0.31)	1.22 (0.94, 1.57)
	Age ≥75 years	0.11 (-0.01, 0.23)	1.26 (0.98, 1.55)	-0.09 (-0.31, 0.08)	0.86 (0.59, 1.14)
Anxiety disorders	Age <55 years	0.05 (-0.03, 0.17)	1.31 (0.85, 2.27)	-0.09 (-0.19, 0.05)	0.73 (0.46, 1.23)
	Age 55-64 years	-0.06 (-0.13, 0.01)	0.73 (0.44, 1.05)	-0.05 (-0.15, 0.04)	0.75 (0.42, 1.21)
	Age 65-74 years	0.04 (-0.05, 0.12)	1.15 (0.83, 1.48)	0.02 (-0.11, 0.12)	1.06 (0.69, 1.48)
	Age ≥75 years	0.10 (0.00, 0.20)	1.29 (0.99, 1.67)	0.13 (-0.03, 0.26)	1.42 (0.91, 2.20)
Self-harm or suicide [b]	Age <55 years	0.01 (-0.04, 0.06)	1.23 (0.27, 3.41)	-0.01 (-0.07, 0.06)	0.85 (0.18, 3.42)
	Age 55-64 years	0.01 (-0.02, 0.05)	1.50 (0.32, 4.95)	0.02 (-0.02, 0.06)	2.10 (0.34, 7.49)
	Age 65-74 years	0.01 (-0.01, 0.04)	1.74 (0.47, 3.95)	0.00 (-0.03, 0.04)	1.10 (0.31, 6.73)
	Age ≥75 years	-0.01 (-0.04, 0.03)	0.87 (0.25, 1.75)	0.00 (-0.05, 0.05)	1.03 (0.21, 5.40)
Any diagnosis of a MHC	Age <55 years	0.64 (0.37, 0.95)	1.35 (1.19, 1.52)	-0.15 (-0.51, 0.31)	0.94 (0.82, 1.13)
	Age 55-64 years	0.62 (0.37, 0.87)	1.39 (1.23, 1.57)	0.23 (-0.10, 0.55)	1.12 (0.95, 1.29)
	Age 65-74 years	0.60 (0.36, 0.84)	1.24 (1.14, 1.34)	0.42 (0.10, 0.77)	1.15 (1.04, 1.30)
	Age ≥75 years	0.67 (0.28, 1.10)	1.09 (1.04, 1.15)	-0.01 (-0.65, 0.66)	1.00 (0.92, 1.09)
**Offspring cohort**					
Depression	Age <35 years	0.13 (0.03, 0.23)	1.18 (1.04, 1.32)	-0.00 (-0.13, 0.11)	1.00 (0.86, 1.13)
	Age 35-44 years	-0.01 (-0.08, 0.05)	0.98 (0.84, 1.09)	-0.02 (-0.10, 0.06)	0.96 (0.82, 1.13)
	Age 45-54 years	0.03 (-0.03, 0.09)	1.06 (0.92, 1.21)	0.02 (-0.06, 0.11)	1.05 (0.88, 1.26)
	Age ≥55 years	-0.05 (-0.12, 0.02)	0.88 (0.71, 1.07)	-0.03 (-0.15, 0.07)	0.91 (0.67, 1.24)
Substance use disorders	Age <35 years	0.13 (0.03, 0.23)	1.20 (1.04, 1.36)	0.01 (-0.09, 0.12)	1.01 (0.89, 1.16)
	Age 35-44 years	0.07 (0.01, 0.15)	1.13 (1.01, 1.28)	-0.02 (-0.11, 0.07)	0.96 (0.84, 1.13)
	Age 45-54 years	0.03 (-0.04, 0.09)	1.05 (0.94, 1.16)	-0.05 (-0.14, 0.04)	0.92 (0.81, 1.07)
	Age ≥55 years	-0.02 (-0.11, 0.10)	0.97 (0.80, 1.19)	0.02 (-0.13, 0.17)	1.04 (0.79, 1.36)
Anxiety disorders	Age <35 years	0.09 (0.02, 0.16)	1.28 (1.07, 1.55)	0.04 (-0.04, 0.13)	1.12 (0.90, 1.39)
	Age 35-44 years	-0.02 (-0.05, 0.02)	0.92 (0.74, 1.13)	-0.01 (-0.06, 0.04)	0.93 (0.70, 1.25)
	Age 45-54 years	0.01 (-0.02, 0.05)	1.09 (0.87, 1.35)	-0.01 (-0.06, 0.05)	0.95 (0.68, 1.33)
	Age ≥55 years	-0.02 (-0.07, 0.04)	0.89 (0.53, 1.25)	0.05 (-0.02, 0.12)	1.60 (0.85, 3.68)
Self-harm or suicide [b]	Age <35 years	-0.00 (-0.03, 0.03)	0.99 (0.62, 1.48)	0.00 (-0.03, 0.03)	1.04 (0.62, 1.81)
	Age 35-44 years	0.01 (-0.01, 0.03)	1.35 (0.80, 2.13)	0.01 (-0.02, 0.03)	1.20 (0.59, 2.62)
	Age 45-54 years	0.02 (0.01, 0.04)	1.93 (1.27, 2.78)	0.02 (-0.00, 0.04)	1.61 (0.92, 3.04)
	Age ≥55 years	0.01 (-0.01, 0.03)	1.82 (0.50, 4.58)	0.00 (-0.02, 0.03)	1.26 (0.31, 7.36)
Any diagnosis of a MHC	Age <35 years	0.49 (0.29, 0.68)	1.17 (1.10, 1.24)	-0.04 (-0.30, 0.16)	0.99 (0.92, 1.05)
	Age 35-44 years	0.05 (-0.07, 0.19)	1.03 (0.96, 1.10)	-0.16 (-0.30, 0.04)	0.93 (0.86, 1.02)
	Age 45-54 years	0.10 (-0.02, 0.20)	1.06 (0.99, 1.12)	-0.01 (-0.18, 0.15)	0.99 (0.90, 1.09)
	Age ≥55 years	-0.06 (-0.25, 0.14)	0.96 (0.85, 1.08)	0.02 (-0.24, 0.26)	1.02 (0.86, 1.18)

Abbreviations: CI: confidence interval; GP: general population; MI: myocardial infarction; MHC: mental health condition.

[a] Risk differences and risk ratios were weighted for the following variables: age and sex of both study participants and their respective partners or parents, year of index date, household income, highest achieved education, comorbidities (32 distinct conditions), comedications, and healthcare utilization (full list in **eTable 2**).

[b] Self-harm or suicide was not included in the composite outcome of any mental health conditions.

© 2024 Skajaa N et al. *JAMA Network Open.*

eTable 7. Associations Between Stroke in a Partner or Parent and Risk of Depression, Substance Use Disorders, Anxiety Disorders, Self-Harm or Suicide, and a Composite Outcome of Any Diagnosis of a Mental Health Condition, Stratified by Sex

		Stroke *vs*. GP		Stroke *vs*. MI	
		**3-year risk difference, % (95% CI)** [a]	**3-year risk ratio (95% CI)** [a]	**3-year risk difference, % (95% CI)** [a]	**3-year risk ratio (95% CI)** [a]
**Partner cohort**					
Depression	Men	0.27 (0.14, 0.40)	1.35 (1.17, 1.55)	0.08 (-0.13, 0.29)	1.08 (0.88, 1.35)
	Women	0.23 (0.14, 0.34)	1.29 (1.17, 1.45)	0.08 (-0.05, 0.24)	1.09 (0.95, 1.27)
Substance use disorders	Men	0.25 (0.11, 0.37)	1.36 (1.16, 1.56)	-0.04 (-0.26, 0.14)	0.96 (0.76, 1.15)
	Women	0.20 (0.12, 0.26)	1.46 (1.26, 1.64)	0.12 (0.02, 0.22)	1.24 (1.05, 1.49)
Anxiety disorders	Men	0.03 (-0.03, 0.10)	1.14 (0.88, 1.53)	0.04 (-0.05, 0.11)	1.17 (0.80, 1.62)
	Women	0.04 (-0.02, 0.09)	1.14 (0.92, 1.35)	0.00 (-0.06, 0.08)	1.00 (0.81, 1.31)
Self-harm or suicide [b]	Men	0.00 (-0.03, 0.03)	1.01 (0.33, 1.94)	-0.02 (-0.07, 0.02)	0.61 (0.20, 1.72)
	Women	0.01 (-0.01, 0.03)	1.41 (0.79, 2.49)	0.02 (-0.00, 0.04)	1.87 (0.94, 5.07)
Any diagnosis of a MHC	Men	0.53 (0.27, 0.76)	1.13 (1.06, 1.19)	0.01 (-0.42, 0.37)	1.00 (0.91, 1.08)
	Women	0.69 (0.50, 0.89)	1.23 (1.16, 1.29)	0.26 (0.00, 0.53)	1.07 (1.00, 1.16)
**Offspring cohort**					
Depression	Men	0.00 (-0.04- 0.05)	1.00 (0.89, 1.12)	-0.04 (-0.10, 0.02)	0.90 (0.80, 1.04)
	Women	0.07 (0.02, 0.13)	1.11 (1.03, 1.21)	0.04 (-0.03, 0.11)	1.06 (0.95, 1.18)
Substance use disorders	Men	0.06 (-0.01, 0.13)	1.09 (0.98, 1.19)	-0.01 (-0.08, 0.07)	0.99 (0.90, 1.09)
	Women	0.07 (0.02, 0.12)	1.14 (1.04, 1.26)	-0.03 (-0.10, 0.04)	0.94 (0.83, 1.08)
Anxiety disorders	Men	0.01 (-0.01, 0.04)	1.09 (0.91, 1.35)	0.01 (-0.02, 0.05)	1.12 (0.88, 1.44)
	Women	0.03 (-0.01, 0.07)	1.10 (0.95, 1.26)	0.01 (-0.05, 0.05)	1.02 (0.84, 1.21)
Self-harm or suicide [b]	Men	0.01 (0.00, 0.03)	1.42 (1.01, 2.06)	0.01 (-0.01, 0.03)	1.17 (0.78, 1.79)
	Women	0.01 (-0.00, 0.03)	1.42 (0.88, 2.03)	0.01 (-0.00, 0.03)	1.45 (0.88, 2.43)
Any diagnosis of a MHC	Men	0.16 (0.06, 0.28)	1.09 (1.03, 1.16)	-0.03 (-0.14, 0.10)	0.99 (0.93, 1.05)
	Women	0.19 (0.08, 0.29)	1.08 (1.03, 1.13)	-0.07 (-0.21, 0.06)	0.97 (0.92, 1.02)

Abbreviations: CI: confidence interval; GP: general population; MI: myocardial infarction; MHC: mental health condition.
[a] Risk differences and risk ratios were weighted for the following variables: age and sex of both study participants and their respective partners or parents, year of index date, household income, highest achieved education, comorbidities (32 distinct conditions), comedications, and healthcare utilization (full list in **eTable 2**).
[b] Self-harm or suicide was not included in the composite outcome of any mental health conditions.

© 2024 Skajaa N et al. *JAMA Network Open.*

eTable 8. Associations Between Stroke in a Partner or Parent and Risk of Depression, Substance Use Disorders, Anxiety Disorders, Self-Harm or Suicide, and a Composite Outcome of Any Diagnosis of a Mental Health Condition, Stratified by Number of Comorbidities

		**Stroke *vs.* GP**		**Stroke *vs.* MI**	
		**3-year risk difference, % (95% CI) [a]**	**3-year risk ratio (95% CI) [a]**	**3-year risk difference, % (95% CI) [a]**	**3-year risk ratio (95% CI) [a]**
**Partner cohort**					
Depression	0 baseline comorbidity	0.18 (0.07, 0.29)	1.48 (1.19- 1.80)	0.18 (0.05, 0.34)	1.47 (1.12, 2.13)
	1 baseline comorbidity	0.31 (0.14, 0.44)	1.55 (1.24, 1.86)	0.06 (-0.17, 0.28)	1.07 (0.81, 1.44)
	2 baseline comorbidities	0.21 (0.02, 0.42)	1.27 (1.03, 1.57)	-0.03 (-0.28, 0.24)	0.97 (0.75, 1.29)
	3 baseline comorbidities	0.39 (0.12, 0.64)	1.42 (1.13, 1.71)	0.06 (-0.33, 0.47)	1.05 (0.78, 1.45)
	≥4 baseline comorbidities	0.23 (0.02, 0.47)	1.16 (1.02, 1.31)	0.09 (-0.21, 0.42)	1.06 (0.89, 1.29)
Substance use disorders	0 baseline comorbidity	0.16 (0.04, 0.25)	1.40 (1.10, 1.68)	0.01 (-0.14, 0.14)	1.02 (0.78, 1.33)
	1 baseline comorbidity	0.17 (0.04, 0.33)	1.39 (1.09, 1.78)	0.01 (-0.16, 0.22)	1.02 (0.78, 1.47)
	2 baseline comorbidities	0.29 (0.12, 0.48)	1.53 (1.21, 1.95)	0.11 (-0.13, 0.40)	1.15 (0.85, 1.70)
	3 baseline comorbidities	0.24 (0.05, 0.44)	1.43 (1.08, 1.84)	0.21 (-0.05, 0.44)	1.35 (0.94, 1.96)
	≥4 baseline comorbidities	0.25 (0.06, 0.40)	1.34 (1.08, 1.62)	0.05 (-0.19, 0.31)	1.06 (0.82, 1.42)
Anxiety disorders	0 baseline comorbidity	0.00 (-0.07, 0.07)	0.96 (0.55, 1.58)	-0.06 (-0.15, 0.02)	0.67 (0.39, 1.13)
	1 baseline comorbidity	0.04 (-0.03, 0.13)	1.23 (0.85, 1.75)	0.04 (-0.06, 0.15)	1.23 (0.73, 2.10)
	2 baseline comorbidities	0.03 (-0.08, 0.15)	1.12 (0.68, 1.77)	-0.07 (-0.19, 0.08)	0.78 (0.45, 1.33)
	3 baseline comorbidities	0.13 (-0.01, 0.29)	1.41 (0.96, 2.06)	0.11 (-0.06, 0.29)	1.35 (0.85, 2.26)
	≥4 baseline comorbidities	0.03 (-0.10, 0.16)	1.07 (0.79, 1.39)	0.07 (-0.09, 0.25)	1.17 (0.80, 1.75)
Self-harm or suicide [b]	0 baseline comorbidity	0.03 (-0.01, 0.06)	2.66 (0.64, 7.57)	0.01 (-0.02, 0.05)	1.48 (0.36, 7.09)
	1 baseline comorbidity	0.01 (-0.02, 0.05)	1.38 (0.43, 3.26)	0.02 (-0.01, 0.06)	2.11 (0.64, 10.40)
	2 baseline comorbidities	0.02 (-0.02, 0.06)	1.83 (0.51,4.40)	0.01 (-0.03, 0.06)	1.48 (0.30, 7.61)
	3 baseline comorbidities	-0.00 (-0.04, 0.04)	0.96 (0.00, 2.73)	-0.03 (-0.12, 0.04)	0.50 (0.00, 2.97)
	≥4 baseline comorbidities	-0.03 (-0.06, 0.00)	0.45 (0.00, 1.08)	-0.01 (-0.06, 0.03)	0.62 (0.00, 3.89)
Any diagnosis of a MHC	0 baseline comorbidity	0.42 (0.20, 0.62)	1.25 (1.11, 1.38)	0.11 (-0.16, 0.40)	1.06 (0.92, 1.22)
	1 baseline comorbidity	0.62 (0.29, 0.87)	1.24 (1.11, 1.35)	0.01 (-0.48, 0.43)	1.00 (0.86, 1.14)
	2 baseline comorbidities	0.77 (0.34, 1.16)	1.23 (1.10, 1.36)	0.18 (-0.30, 0.72)	1.05 (0.93, 1.20)
	3 baseline comorbidities	1.10 (0.62, 1.60)	1.28 (1.15, 1.41)	0.48 (-0.20, 1.17)	1.10 (0.96, 1.28)
	≥4 baseline comorbidities	0.65 (0.17, 1.12)	1.11 (1.03, 1.18)	0.09 (-0.55, 0.73)	1.01 (0.92, 1.11)
**Offspring cohort**					
Depression	0 baseline comorbidity	0.02 (-0.02, 0.07)	1.05 (0.95, 1.16)	-0.00 (-0.06, 0.06)	0.99 (0.87, 1.14)
	1 baseline comorbidity	0.09 (0.02, 0.17)	1.16 (1.03, 1.33)	0.04 (-0.05, 0.14)	1.06 (0.92, 1.26)
	2 baseline comorbidities	-0.01 (-0.17, 0.13)	0.99 (0.79, 1.18)	-0.10 (-0.28, 0.10)	0.88 (0.70, 1.14)
	3 baseline comorbidities	0.10 (-0.10, 0.36)	1.12 (0.87, 1.44)	0.12 (-0.25, 0.44)	1.14 (0.76, 1.74)
	≥4 baseline comorbidities	-0.18 (-0.55, 0.22)	0.86 (0.60, 1.18)	-0.30 (-0.81, 0.15)	0.79 (0.52, 1.15)
Substance use disorders	0 baseline comorbidity	0.08 (0.04, 0.11)	1.17 (1.08, 1.25)	0.04 (-0.03, 0.10)	1.07 (0.95, 1.21)
	1 baseline comorbidity	0.11 (0.01, 0.19)	1.17 (1.02, 1.30)	-0.06 (-0.19, 0.06)	0.93 (0.78, 1.08)
	2 baseline comorbidities	-0.01 (-0.15, 0.16)	0.99 (0.81, 1.22)	-0.13 (-0.34, 0.15)	0.85 (0.65, 1.20)
	3 baseline comorbidities	-0.19 (-0.41, 0.08)	0.80 (0.59, 1.09)	-0.31 (-0.63, 0.04)	0.71 (0.50, 1.04)
	≥4 baseline comorbidities	-0.12 (-0.50, 0.22)	0.91 (0.63, 1.18)	0.04 (-0.39, 0.45)	1.04 (0.69, 1.48)
Anxiety disorders	0 baseline comorbidity	0.01 (-0.02, 0.04)	1.07 (0.89, 1.28)	0.00 (-0.04, 0.04)	0.98 (0.79, 1.26)
	1 baseline comorbidity	0.06 (0.00, 0.10)	1.28 (1.01, 1.54)	0.05 (-0.02, 0.12)	1.25 (0.92, 1.66)
	2 baseline comorbidities	0.01 (-0.07, 0.10)	1.03 (0.74, 1.41)	0.02 (-0.08, 0.13)	1.06 (0.73, 1.68)
	3 baseline comorbidities	0.02 (-0.12, 0.18)	1.06 (0.60, 1.67)	0.07 (-0.13, 0.24)	1.26 (0.67, 2.40)
	≥4 baseline comorbidities	-0.13 (-0.30, 0.03)	0.70 (0.34, 1.09)	-0.22 (-0.55, 0.06)	0.57 (0.26, 1.26)
Self-harm or suicide [b]	0 baseline comorbidity	0.01 (-0.00, 0.02)	1.34 (0.93-1.80)	0.01 (-0.00, 0.03)	1.48 (0.93, 2.63)
	1 baseline comorbidity	0.01 (-0.01, 0.03)	1.30 (0.81-1.98)	0.00 (-0.02, 0.03)	1.06 (0.59, 1.81)
	2 baseline comorbidities	0.01 (-0.02, 0.05)	1.21 (0.50-2.48)	0.02 (-0.02, 0.06)	1.53 (0.55, 5.60)
	3 baseline comorbidities	0.06 (-0.01, 0.16)	2.13 (0.77-4.91)	0.05 (-0.07, 0.15)	1.76 (0.47, 7.43)
	≥4 baseline comorbidities	0.08 (-0.00, 0.20)	4.04 (0.91-15.94)	-0.04 (-0.24, 0.12)	0.74 (0.13, 2.87)
Any diagnosis of a MHC	0 baseline comorbidity	0.19 (0.10, 0.28)	1.12 (1.06, 1.17)	-0.00 (-0.12, 0.09)	1.00 (0.94, 1.05)
	1 baseline comorbidity	0.31 (0.16, 0.47)	1.14 (1.07, 1.21)	0.03 (-0.14, 0.23)	1.01 (0.94, 1.10)
	2 baseline comorbidities	0.01 (-0.31, 0.27)	1.00 (0.89, 1.10)	-0.33 (-0.71, 0.04)	0.89 (0.79, 1.01)
	3 baseline comorbidities	-0.19 (-0.59, 0.32)	0.94 (0.83, 1.10)	-0.15 (-0.79, 0.62)	0.95 (0.78, 1.22)
	≥4 baseline comorbidities	-0.70 (-1.29, -0.11)	0.84 (0.71, 0.98)	-0.55 (-1.46, 0.02)	0.87 (0.70, 1.00)

Abbreviations: CI: confidence interval; GP: general population; MI: myocardial infarction; MHC: mental health condition.

© 2024 Skajaa N et al. *JAMA Network Open.*

[a] Risk differences and risk ratios were weighted for the following variables: age and sex of both study participants and their respective partners or parents, year of index date, household income, highest achieved education, comorbidities (32 distinct conditions), comedications, and healthcare utilization (full list in **eTable 2**).
[b] Self-harm or suicide was not included in the composite outcome of any mental health conditions.

eTable 9. Associations Between Stroke in a Partner or Parent and Risk of Depression, Substance Use Disorders, Anxiety Disorders, Self-Harm or Suicide, and a Composite Outcome of Any Diagnosis of a Mental Health Condition, Stratified by Household Income

		Stroke *vs.* GP		Stroke *vs.* MI	
		**3-year risk difference, % (95% CI) [a]**	**3-year risk ratio (95% CI) [a]**	**3-year risk difference, % (95% CI) [a]**	**3-year risk ratio (95% CI) [a]**
**Partner cohort**					
Depression	Low household income	0.34 (0.18, 0.51)	1.33 (1.17, 1.50)	0.01 (-0.20, 0.23)	1.00 (0.86, 1.18)
	Medium household income	0.18 (0.06, 0.28)	1.26 (1.08, 1.41)	0.15 (0.00, 0.29)	1.20 (1.00, 1.43)
	High household income	0.23 (0.08, 0.40)	1.42 (1.15, 1.74)	0.05 (-0.22, 0.33)	1.06 (0.78, 1.59)
Substance use disorders	Low household income	0.29 (0.13, 0.46)	1.45 (1.18, 1.75)	0.16 (-0.02, 0.32)	1.20 (0.97, 1.45)
	Medium household income	0.22 (0.13, 0.34)	1.44 (1.25, 1.70)	-0.01 (-0.14, 0.13)	0.98 (0.83, 1.20)
	High household income	0.06 (-0.05, 0.20)	1.19 (0.87, 1.59)	0.07 (-0.10, 0.22)	1.20 (0.77, 1.88)
Anxiety disorders	Low household income	-0.01 (-0.10, 0.07)	0.96 (0.69, 1.24)	-0.07 (-0.17, 0.02)	0.81 (0.61, 1.07)
	Medium household income	0.04 (-0.04, 0.12)	1.16 (0.83, 1.49)	0.03 (-0.06, 0.12)	1.11 (0.80, 1.62)
	High household income	0.11 (0.02, 0.20)	1.64 (1.13, 2.32)	0.12 (0.00, 0.24)	1.78 (1.01, 3.33)
Self-harm or suicide [b]	Low household income	0.02 (-0.01, 0.05)	1.53 (0.73, 2.90)	0.01 (-0.02, 0.05)	1.27 (0.50, 4.16)
	Medium household income	-0.00 (-0.02, 0.02)	0.89 (0.23, 1.75)	-0.01 (-0.04, 0.02)	0.67 (0.15, 2.07)
	High household income	0.01 (-0.02, 0.05)	1.46 (0.35, 3.60)	0.02 (-0.02, 0.06)	2.63 (0.51, 10.70)
Any diagnosis of a MHC	Low household income	0.78 (0.48, 1.11)	1.18 (1.11, 1.26)	0.07 (-0.35, 0.45)	1.01 (0.93, 1.09)
	Medium household income	0.63 (0.38, 0.89)	1.21 (1.12, 1.30)	0.24 (-0.12, 0.58)	1.07 (0.97, 1.18)
	High household income	0.48 (0.15, 0.84)	1.18 (1.05, 1.33)	0.05 (-0.43, 0.47)	1.02 (0.87, 1.16)
**Offspring cohort**					
Depression	Low household income	0.08 (-0.05- 0.20)	1.10 (0.95, 1.25)	0.09 (-0.08, 0.24)	1.11 (0.91, 1.33)
	Medium household income	0.05 (-0.01, 0.11)	1.10 (0.98, 1.20)	0.04 (-0.04, 0.10)	1.07 (0.94, 1.19)
	High household income	-0.02 (-0.06, 0.03)	0.92 (0.80, 1.10)	-0.11 (-0.18, -0.04)	0.72 (0.59, 0.88)
Substance use disorders	Low household income	0.00 (-0.13, 0.15)	1.00 (0.89, 1.13)	-0.33 (-0.51, -0.12)	0.78 (0.69, 0.91)
	Medium household income	0.09 (0.02, 0.14)	1.16 (1.04, 1.25)	0.06 (-0.01, 0.14)	1.11 (0.98, 1.25)
	High household income	0.03 (-0.01, 0.08)	1.11 (0.97, 1.28)	-0.01 (-0.08, 0.05)	0.96 (0.78, 1.17)
Anxiety disorders	Low household income	0.08 (0.01, 0.16)	1.26 (1.02, 1.53)	0.05 (-0.05, 0.15)	1.15 (0.89, 1.49)
	Medium household income	0.01 (-0.02, 0.04)	1.06 (0.90, 1.19)	0.01 (-0.04, 0.05)	1.06 (0.84, 1.28)
	High household income	0.00 (-0.03, 0.03)	0.98 (0.73, 1.25)	-0.01 (-0.06, 0.03)	0.89 (0.63, 1.25)
Self-harm or suicide [b]	Low household income	0.03 (-0.01, 0.06)	1.64 (0.88, 2.76)	0.00 (-0.04, 0.05)	1.02 (0.52, 2.16)
	Medium household income	0.01 (-0.00, 0.03)	1.31 (0.90, 1.84)	0.02 (-0.00, 0.03)	1.48 (0.95, 2.31)
	High household income	0.01 (-0.01, 0.02)	1.46 (0.74, 2.38)	0.00 (-0.02-0.02)	1.15 (0.51, 2.48)
Any diagnosis of a MHC	Low household income	0.40 (0.14, 0.61)	1.12 (1.04, 1.18)	-0.19 (-0.46, 0.10)	0.95 (0.89, 1.02)
	Medium household income	0.14 (0.03, 0.22)	1.07 (1.01, 1.11)	0.04 (-0.10, 0.17)	1.02 (0.96, 1.08)
	High household income	0.10 (0.02, 0.19)	1.08 (1.01, 1.16)	-0.13 (-0.26, 0.03)	0.91 (0.83, 1.02)

Abbreviations: CI: confidence interval; GP: general population; MI: myocardial infarction; MHC: mental health condition.

[a] Risk differences and risk ratios were weighted for the following variables: age and sex of both study participants and their respective partners or parents, year of index date, household income, highest achieved education, comorbidities (32 distinct conditions), comedications, and healthcare utilization (full list in **eTable 2**).

[b] Self-harm or suicide was not included in the composite outcome of any mental health conditions.

© 2024 Skajaa N et al. *JAMA Network Open.*

eTable 10. Associations Between Stroke in a Partner or Parent and Risk of Depression, Substance Use Disorders, Anxiety Disorders, Self-Harm or Suicide, and a Composite Outcome of Any Diagnosis of a Mental Health Condition, Stratified by Highest Achieved Education

		**Stroke *vs.* GP**		**Stroke *vs.* MI**	
		**3-year risk difference, % (95% CI) [a]**	**3-year risk ratio (95% CI) [a]**	**3-year risk difference, % (95% CI) [a]**	**3-year risk ratio (95% CI) [a]**
**Partner cohort**					
Depression	Low education	0.26 (0.12, 0.40)	1.28 (1.12, 1.45)	0.10 (-0.05, 0.30)	1.09 (0.95, 1.30)
	Medium education	0.21 (0.08, 0.35)	1.30 (1.12, 1.51)	0.05 (-0.10, 0.21)	1.05 (0.90, 1.27)
	High education	0.26 (0.07, 0.43)	1.45 (1.13, 1.77)	0.12 (-0.10, 0.33)	1.17 (0.87, 1.57)
Substance use disorders	Low education	0.22 (0.10, 0.34)	1.38 (1.16, 1.61)	0.10 (-0.04, 0.28)	1.15 (0.95, 1.45)
	Medium education	0.26 (0.13, 0.38)	1.48 (1.24, 1.76)	0.06 (-0.12, 0.22)	1.09 (0.85, 1.34)
	High education	0.10 (-0.01, 0.24)	1.26 (0.97, 1.71)	-0.04 (-0.20, 0.13)	0.92 (0.66, 1.34)
Anxiety disorders	Low education	-0.01 (-0.10, 0.07)	0.97 (0.70, 1.23)	-0.03 (-0.15, 0.07)	0.92 (0.65, 1.26)
	Medium education	0.07 (-0.02, 0.13)	1.30 (0.92, 1.66)	0.03 (-0.08, 0.13)	1.11 (0.75, 1.67)
	High education	0.05 (-0.03, 0.14)	1.25 (0.82, 1.74)	0.05 (-0.07, 0.15)	1.24 (0.73, 2.05)
Self-harm or suicide [b]	Low education	0.01 (-0.02, 0.03)	1.20 (0.37, 2.41)	0.02 (-0.01, 0.05)	2.26 (0.71, 15.63)
	Medium education	0.01 (-0.02, 0.03)	1.21 (0.48, 2.77)	-0.01 (-0.05, 0.02)	0.72 (0.26, 2.05)
	High education	-0.00 (-0.04, 0.03)	0.96 (0.00, 2.25)	-0.02 (-0.08, 0.03)	0.68 (0.00, 3.21)
Any diagnosis of a MHC	Low education	0.73 (0.43, 1.01)	1.18 (1.10, 1.25)	0.30 (-0.08, 0.65)	1.07 (0.98, 1.15)
	Medium education	0.70 (0.46, 0.96)	1.24 (1.15, 1.33)	0.13 (-0.25, 0.46)	1.04 (0.93, 1.14)
	High education	0.62 (0.30, 0.92)	1.25 (1.11, 1.37)	0.18 (-0.32, 0.66)	1.06 (0.90, 1.25)
**Offspring cohort**					
Depression	Low education	0.13 (0.01, 0.22)	1.16 (1.01, 1.28)	0.06 (-0.07, 0.20)	1.07 (0.92, 1.25)
	Medium education	0.02 (-0.03, 0.08)	1.05 (0.94, 1.16)	-0.04 (-0.11, 0.04)	0.93 (0.82, 1.07)
	High education	-0.02 (-0.07, 0.04)	0.96 (0.83, 1.10)	0.01 (-0.06, 0.08)	1.03 (0.85, 1.26)
Substance use disorders	Low education	0.04 (-0.07, 0.18)	1.04 (0.94, 1.15)	-0.12 (-0.26, 0.05)	0.91 (0.81, 1.04)
	Medium education	0.07 (0.01, 0.13)	1.13 (1.02, 1.24)	0.01 (-0.06, 0.09)	1.02 (0.91, 1.17)
	High education	0.02 (-0.02, 0.07)	1.08 (0.93, 1.28)	-0.01 (-0.07, 0.06)	0.96 (0.79, 1.22)
Anxiety disorders	Low education	0.06 (-0.01, 0.13)	1.20 (0.96, 1.46)	0.11 (0.02, 0.19)	1.43 (1.06, 1.86)
	Medium education	0.03 (0.00, 0.07)	1.19 (0.98, 1.40)	0.00 (-0.04, 0.05)	1.01 (0.79, 1.27)
	High education	-0.04 (-0.06, -0.01)	0.76 (0.58, 0.96)	-0.06 (-0.10, -0.01)	0.67 (0.46, 0.91)
Self-harm or suicide [b]	Low education	0.02 (-0.01, 0.06)	1.32 (0.82, 1.88)	0.03 (-0.01, 0.07)	1.48 (0.90, 2.57)
	Medium education	0.01 (-0.00, 0.03)	1.43 (0.99, 2.03)	-0.00 (-0.02, 0.02)	0.98 (0.61, 1.55)
	High education	0.01 (-0.00, 0.02)	1.66 (0.72, 2.89)	0.01 (-0.01, 0.03)	1.63 (0.65, 7.64)
Any diagnosis of a MHC	Low education	0.36 (0.14, 0.58)	1.11 (1.04, 1.17)	-0.06 (-0.29, 0.25)	0.98 (0.92, 1.07)
	Medium education	0.16 (0.06, 0.25)	1.09 (1.03, 1.14)	-0.09 (-0.21, 0.05)	0.96 (0.90, 1.02)
	High education	-0.01 (-0.10, 0.10)	0.99 (0.93, 1.07)	-0.02 (-0.18, 0.12)	0.99 (0.89, 1.09)

Abbreviations: CI: confidence interval; GP: general population; MI: myocardial infarction; MHC: mental health condition.

[a] Risk differences and risk ratios were weighted for the following variables: age and sex of both study participants and their respective partners or parents, year of index date, household income, highest achieved education, comorbidities (32 distinct conditions), comedications, and healthcare utilization (full list in **eTable 2**).

[b] Self-harm or suicide was not included in the composite outcome of any mental health conditions.

© 2024 Skajaa N et al. *JAMA Network Open.*

eTable 11. Numbers of Events and 3-Year Absolute Risks of Depression (Defined From Either a Hospital-Based Diagnosis or ≥2 Prescriptions for an Antidepressant With Indication Code for Depression), Depression (Additionally Including Persistent Mood Disorders), and Nonmelanoma Skin Cancer

	Events, N [a]	3-year absolute risk, % [a]
**Partner cohort**		
Depression (diagnosis or ≥2 prescriptions with indication)		
Stroke	5979	8.98
GP	23545	7.07
MI	5766	8.59
Depression (additionally including persistent mood disorders)		
Stroke	681	1.06
GP	2419	0.75
MI	611	0.93
Non-melanoma skin cancer		
Stroke	610	0.97
GP	3189	1.01
MI	505	0.78
**Offspring cohort**		
Depression (diagnosis or ≥2 prescriptions with indication)		
Stroke	9207	4.76
GP	42543	4.4
MI	8524	4.92
Depression (additionally including persistent mood disorders)		
Stroke	1060	0.56
GP	4726	0.50
MI	1050	0.62
Non-melanoma skin cancer		
Stroke	336	0.18
GP	1730	0.19
MI	244	0.15

Abbreviations: CI: confidence interval; GP: general population; MI: myocardial infarction.
[a] Numbers of events and absolute risks were calculated in the original, unweighted population.

© 2024 Skajaa N et al. *JAMA Network Open.*

eTable 12. Associations Between Stroke in a Partner or Parent and Risk of Depression (Defined From Either a Hospital-Based Diagnosis or ≥2 Prescriptions for an Antidepressant With Indication Code for Depression), Depression (Additionally Including Persistent Mood Disorders), and Nonmelanoma Skin Cancer

	**Stroke *vs*. GP**		**Stroke *vs*. MI**	
	**3-year risk difference, % (95% CI)** [a]	**3-year risk ratio (95% CI)** [a]	**3-year risk difference, % (95% CI)** [a]	**3-year risk ratio (95% CI)** [a]
**Partner cohort**				
Depression (diagnosis or ≥2 prescriptions with indication)	1.42 (1.18, 1.59)	1.19 (1.16, 1.21)	0.41 (0.12, 0.66)	1.05 (1.01, 1.08)
Depression (additionally including persistent mood disorders)	0.25 (0.16, 0.32)	1.31 (1.19, 1.41)	0.09 (-0.02, 0.21)	1.09 (0.98, 1.24)
Non-melanoma skin cancer	-0.02 (-0.10, 0.06)	0.98 (0.90, 1.06)	-0.02 (-0.13, 0.10)	0.98 (0.88, 1.11)
**Offspring cohort**				
Depression (diagnosis or ≥2 prescriptions with indication)	0.17 (0.07, 0.24)	1.04 (1.01, 1.05)	-0.09 (-0.23, 0.02)	0.98 (0.95, 1.01)
Depression (additionally including persistent mood disorders)	0.03 (-0.01, 0.07)	1.07 (0.99, 1.14)	-0.01 (-0.06, 0.05)	0.99 (0.90, 1.09)
Non-melanoma skin cancer	-0.01 (-0.02, 0.02)	0.97 (0.87, 1.08)	0.00 (-0.03, 0.03)	1.01 (0.86, 1.19)

Abbreviations: CI: confidence interval; GP: general population; MI: myocardial infarction.
[a] Risk differences and risk ratios were weighted for the following variables: age and sex of both study participants and their respective partners or parents, year of index date, household income, highest achieved education, comorbidities (32 distinct conditions), comedications, and healthcare utilization (full list in **eTable 2**).

© 2024 Skajaa N et al. *JAMA Network Open.*

eTable 13. Associations Between Stroke in a Partner or Parent and Risk of Depression, Substance Use Disorders, Anxiety Disorders, Self-Harm or Suicide, and a Composite Outcome of Any Diagnosis of a Mental Health Condition, When Performing a Complete-Case Analysis in Which Individuals With Missing Data on Household Income or Highest Achieved Education Were Excluded

	Stroke *vs.* GP		Stroke *vs.* MI	
	**3-year risk difference, % (95% CI) [a]**	**3-year risk ratio (95% CI) [a]**	**3-year risk difference, % (95% CI) [a]**	**3-year risk ratio (95% CI) [a]**
**Partner cohort**				
Depression	0.23 (0.16, 0.32)	1.31 (1.20, 1.42)	0.08 (-0.04, 0.19)	1.09 (0.96, 1.22)
Substance use disorders	0.22 (0.15, 0.29)	1.42 (1.28, 1.59)	0.05 (-0.05, 0.15)	1.08 (0.94, 1.23)
Anxiety disorders	0.03 (-0.01, 0.08)	1.13 (0.98, 1.32)	0.01 (-0.05, 0.08)	1.04 (0.85, 1.32)
Self-harm or suicide [b]	0.00 (-0.01, 0.02)	1.15 (0.74, 1.90)	-0.00 (-0.02, 0.02)	1.00 (0.50, 1.90)
Any diagnosis of a MHC	0.68 (0.54, 0.83)	1.21 (1.16, 1.26)	0.20 (-0.02, 0.41)	1.05 (1.00, 1.11)
**Offspring cohort**				
Depression	0.04 (0.00, 0.07)	1.07 (1.00, 1.14)	-0.00 (-0.05, 0.04)	1.00 (0.91, 1.08)
Substance use disorders	0.05 (0.02, 0.09)	1.10 (1.03, 1.16)	-0.02 (-0.08, 0.03)	0.96 (0.89, 1.05)
Anxiety disorders	0.02 (-0.01, 0.04)	1.09 (0.96, 1.23)	0.01 (-0.03, 0.03)	1.03 (0.89, 1.16)
Self-harm or suicide [b]	0.01 (0.00, 0.02)	1.43 (1.09, 1.74)	0.01 (-0.00, 0.02)	1.26 (0.90, 1.74)
Any diagnosis of a MHC	0.16 (0.08, 0.24)	1.08 (1.04, 1.12)	-0.06 (-0.15, 0.03)	0.97 (0.93, 1.01)

Abbreviations: CI: confidence interval; GP: general population; MI: myocardial infarction; MHC: mental health condition.

[a] Risk differences and risk ratios were weighted for the following variables: age and sex of both study participants and their respective partners or parents, year of index date, household income, highest achieved education, comorbidities (32 distinct conditions), comedications, and healthcare utilization (full list in **eTable 2**).

[b] Self-harm or suicide was not included in the composite outcome of any mental health conditions.

© 2024 Skajaa N et al. *JAMA Network Open.*

eTable 14. Associations Between Stroke in a Partner or Parent and Risk of Depression, Substance Use Disorders, Anxiety Disorders, Self-Harm or Suicide, and a Composite Outcome of Any Diagnosis of a Mental Health Condition, When Performing an Analysis Setting the Index Date to the Stroke Admission Date Instead of the Discharge Date

	**Stroke *vs.* GP**		**Stroke *vs.* MI**	
	**3-year risk difference, % (95% CI)** [a]	**3-year risk ratio (95% CI)** [a]	**3-year risk difference, % (95% CI)** [a]	**3-year risk ratio (95% CI)** [a]
**Partner cohort**				
Depression	0.25 (0.16, 0.34)	1.29 (1.19, 1.41)	0.10 (-0.01, 0.22)	1.10 (0.99, 1.23)
Substance use disorders	0.25 (0.17, 0.33)	1.50 (1.34, 1.67)	0.06 (-0.03, 0.17)	1.09 (0.96, 1.27)
Anxiety disorders	0.03 (-0.01, 0.07)	1.12 (0.96, 1.28)	0.02 (-0.03, 0.07)	1.07 (0.88, 1.29)
Self-harm or suicide [b]	0.02 (0.01, 0.04)	2.26 (1.53, 3.50)	0.01 (-0.01, 0.03)	1.14 (0.69, 2.00)
Any diagnosis of a MHC	0.65 (0.50, 0.79)	1.18 (1.14, 1.22)	0.15 (-0.05, 0.37)	1.04 (0.99, 1.09)
**Offspring cohort**				
Depression	0.04 (-0.00, 0.07)	1.08 (1.00, 1.15)	-0.01 (-0.05, 0.04)	0.99 (0.91, 1.07)
Substance use disorders	0.07 (0.03, 0.10)	1.12 (1.05, 1.18)	-0.01 (-0.06, 0.04)	0.98 (0.90, 1.06)
Anxiety disorders	0.01 (-0.01, 0.04)	1.07 (0.95, 1.20)	0.01 (-0.02, 0.04)	1.04 (0.89, 1.20)
Self-harm or suicide [b]	0.01 (0.00, 0.02)	1.34 (1.05, 1.64)	0.01 (-0.00, 0.02)	1.26 (0.92, 1.71)
Any diagnosis of a MHC	0.17 (0.10, 0.23)	1.08 (1.05, 1.11)	-0.04 (-0.12, 0.05)	0.98 (0.95, 1.02)

Abbreviations: CI: confidence interval; GP: general population; MI: myocardial infarction; MHC: mental health condition.
[a] Risk differences and risk ratios were weighted for the following variables: age and sex of both study participants and their respective partners or parents, year of index date, household income, highest achieved education, comorbidities (32 distinct conditions), comedications, and healthcare utilization (full list in **eTable 2**).
[b] Self-harm or suicide was not included in the composite outcome of any mental health conditions.

© 2024 Skajaa N et al. *JAMA Network Open.*

eTable 15. Baseline Characteristics (N, %) of Adult Children of Stroke Patients, Adult Children of Individuals From the General Population, and Adult Children of Myocardial Infarction Patients Both Before and After Propensity Score Weighting

	Unweighted cohorts			PS-weighted cohorts			
	**Stroke-offspring cohort**	**GP-offspring cohort**	**MI-offspring cohort**	**GP-offspring cohort**	**SMD**	**MI- offspring cohort**	**SMD**
**Overall**	207386	1036886	183309	207503	-	207477	-
**Age, years (median, IQR)**	45 (36, 52)	45 (36, 52)	42 (33, 49)	45 (36, 52)	0.00	45 (36, 52)	0.00
**Age of parent, years (median, IQR)**	73 (65, 80)	72 (64, 79)	70 (62, 78)	73 (65, 81)	0.00	73 (65, 80)	0.00
**Sex, n (%)**							
Women	99382 (48)	496875 (48)	88078 (48)	99432 (48)	0.00	99384 (48)	0.00
Men	108004 (52)	540011 (52)	95231 (52)	108071 (52)	0.00	108093 (52)	0.00
**Sex of partner, n (%)**							
Women	92590 (45)	462906 (45)	66195 (36)	92013 (44)	0.00	93038 (45)	0.01
Men	114796 (55)	573980 (55)	117114 (64)	115490 (56)	0.00	114439 (55)	-0.01
**Household income, n (%)**							
Low (<25th percentile)	31008 (15)	145512 (14)	28224 (15)	31070 (15)	0.00	31023 (15)	0.00
Medium (25th-<75th percentile)	110061 (53)	549790 (53)	99375 (54)	110150 (53)	0.00	110285 (53)	0.00
High (≥75th percentile)	65733 (32)	338863 (33)	55286 (30)	65699 (32)	0.00	65576 (32)	0.00
**Highest achieved education, n (%)**							
Low (ISCED level 1-2)	41317 (20)	183351 (18)	41508 (23)	41378 (20)	0.00	41523 (20)	0.00
Medium (ISCED level 3)	94074 (45)	468466 (45)	84121 (46)	94148 (45)	0.00	94247 (45)	0.00
High (ISCED level 5-8)	69034 (33)	371347 (36)	54957 (30)	69018 (33)	0.00	68739 (33)	0.00
**Comorbidity, n (%)**							
Hypertension	20659 (10)	93212 (9)	16034 (9)	20757 (10)	0.00	20786 (10)	0.00
Dyslipidemia	9293 (4)	39934 (4)	7392 (4)	9340 (5)	0.00	9365 (5)	0.00
Ischemic heart disease	3712 (2)	14662 (1)	3164 (2)	3744 (2)	0.00	3742 (2)	0.00
Atrial fibrillation	1531 (1)	7322 (1)	1158 (1)	1540 (1)	0.00	1533 (1)	0.00
Heart failure	556 (0)	2520 (0)	499 (0)	559 (0)	0.00	559 (0)	0.00
Peripheral artery disease	1205 (1)	5419 (1)	937 (1)	1214 (1)	0.00	1221 (1)	0.00
Venous thromboembolism	1798 (1)	8214 (1)	1374 (1)	1805 (1)	0.00	1800 (1)	0.00
Stroke	1899 (1)	7782 (1)	1351 (1)	1908 (1)	0.00	1918 (1)	0.00
Diabetes mellitus	5108 (2)	23467 (2)	4372 (2)	5125 (2)	0.00	5134 (2)	0.00
Thyroid disorders	4626 (2)	23026 (2)	3753 (2)	4645 (2)	0.00	4633 (2)	0.00
Gout	627 (0)	3044 (0)	536 (0)	629 (0)	0.00	635 (0)	0.00
Obstructive pulmonary disease	11976 (6)	59659 (6)	10931 (6)	11991 (6)	0.00	11946 (6)	0.00
Ulcer/chronic gastritis	1520 (1)	7107 (1)	1358 (1)	1524 (1)	0.00	1515 (1)	0.00
Chronic liver disease	721 (0)	3386 (0)	588 (0)	721 (0)	0.00	721 (0)	0.00
Inflammatory bowel disease	2427 (1)	11654 (1)	2024 (1)	2428 (1)	0.00	2414 (1)	0.00
Diverticular disease	1586 (1)	7729 (1)	1106 (1)	1597 (1)	0.00	1597 (1)	0.00
Chronic kidney disease	548 (0)	2763 (0)	516 (0)	551 (0)	0.00	544 (0)	0.00
Prostate disorders	1024 (0)	4934 (0)	660 (0)	1032 (0)	0.00	1029 (0)	0.00
Connective tissue disorders	3102 (1)	14493 (1)	2556 (1)	3108 (1)	0.00	3118 (2)	0.00
Osteoporosis	30449 (15)	146702 (14)	27657 (15)	30474 (15)	0.00	30417 (15)	0.00
HIV/AIDS	146 (0)	674 (0)	103 (0)	147 (0)	0.00	149 (0)	0.00

© 2024 Skajaa N et al. *JAMA Network Open.*

Anemias	389 (0)	1961 (0)	402 (0)	389 (0)	0.00	386 (0)	0.00
Cancer	3134 (2)	16067 (2)	2181 (1)	3146 (2)	0.00	3136 (2)	0.00
Vision problems	777 (0)	3682 (0)	519 (0)	781 (0)	0.00	791 (0)	0.00
Hearing problems	4263 (2)	20470 (2)	3305 (2)	4280 (2)	0.00	4244 (2)	0.00
Migraine	3551 (2)	18169 (2)	2887 (2)	3555 (2)	0.00	3547 (2)	0.00
Epilepsy	10378 (5)	49617 (5)	8874 (5)	10419 (5)	0.00	10397 (5)	0.00
Parkinson's disease	56 (0)	329 (0)	35 (0)	56 (0)	0.00	53 (0)	0.00
Multiple sclerosis	703 (0)	3550 (0)	587 (0)	706 (0)	0.00	703 (0)	0.00
Neuropathies	1713 (1)	8156 (1)	1439 (1)	1714 (1)	0.00	1720 (1)	0.00
Allergy	8943 (4)	45579 (4)	7448 (4)	8954 (4)	0.00	8912 (4)	0.00
Painful condition	5806 (3)	26717 (3)	4637 (3)	5832 (3)	0.00	5860 (3)	0.00
**Use of medications, n (%)**							
Antidepressants	10015 (5)	47285 (5)	8650 (5)	10038 (5)	0.00	10062 (5)	0.00
Anti-dementia drugs	<5	38 (0)	<5	<5	0.00	<5	0.00
Drugs used in addictive disorders	717 (0)	3222 (0)	559 (0)	721 (0)	0.00	713 (0)	0.00
Antipsychotics	873 (0)	3969 (0)	765 (0)	875 (0)	0.00	886 (0)	0.00
Anxiolytics	3103 (1)	14535 (1)	2650 (1)	3108 (1)	0.00	3131 (2)	0.00
Hypnotics and sedatives	5226 (3)	24802 (2)	4216 (2)	5250 (3)	0.00	5259 (3)	0.00
Antiplatelets	5057 (2)	20285 (2)	4136 (2)	5091 (2)	0.00	5102 (2)	0.00
Anticoagulants	1369 (1)	6348 (1)	1028 (1)	1376 (1)	0.00	1366 (1)	0.00
NSAIDs	34335 (17)	163560 (16)	30039 (16)	34409 (17)	0.00	34410 (17)	0.00
Systemic glucocorticoids	5215 (3)	25682 (2)	4720 (3)	5226 (3)	0.00	5235 (3)	0.00
Proton pump inhibitors	13426 (6)	63543 (6)	11198 (6)	13475 (6)	0.00	13459 (6)	0.00
**Number of inpatient admissions in previous 3 years, n (%)**							
0	164945 (80)	834498 (80)	144131 (79)	165032 (80)	0.00	164940 (79)	0.00
1	29033 (14)	140169 (14)	26571 (14)	29056 (14)	0.00	29074 (14)	0.00
≥2	13408 (6)	62219 (6)	12607 (7)	13414 (6)	0.00	13463 (6)	0.00
**Number of outpatient visits in previous 3 years, n (%)**							
0	95739 (46)	487068 (47)	87066 (47)	95723 (46)	0.00	95761 (46)	0.00
1-3	58641 (28)	291576 (28)	50315 (27)	58692 (28)	0.00	58575 (28)	0.00
4-7	26358 (13)	128606 (12)	22182 (12)	26401 (13)	0.00	26395 (13)	0.00
≥8	26648 (13)	129636 (13)	23746 (13)	26688 (13)	0.00	26747 (13)	0.00
**Number of redeemed prescriptions in previous 3 years, n (%)**							
0-5	109966 (53)	562485 (54)	98026 (53)	109944 (53)	0.00	109876 (53)	0.00
6-15	47398 (23)	236719 (23)	42945 (23)	47403 (23)	0.00	47365 (23)	0.00
16-35	31624 (15)	152598 (15)	27320 (15)	31669 (15)	0.00	31741 (15)	0.00
≥36	18398 (9)	85084 (8)	15018 (8)	18487 (9)	0.00	18495 (9)	0.00
**Stroke severity according to the Scandinavian Stroke Scale, n (%)**							
Mild (SSS score 43-58)	141307 (68)	-	-	-	-	-	-
Moderate (SSS score 26-42)	33345 (16)	-	-	-	-	-	-
Severe or very severe (SSS score 0-25)	20105 (10)	-	-	-	-	-	-

© 2024 Skajaa N et al. *JAMA Network Open.*

Missing	12629 (6)	-	-	-	-	-	-
**Stroke subtype, n (%)**							
Ischemic stroke	188344 (91)	-	-	-	-	-	-
Intracerebral hemorrhage	19042 (9)	-	-	-	-	-	-

Abbreviations: GP: general population; MI: myocardial infarction; PS: propensity score; SMD: standardized mean difference; IQR: interquartile range; ISCED: International Standard Classification of Education; NSAID: non-steroidal anti-inflammatory drugs; SSS: Scandinavian Stroke Scale.

© 2024 Skajaa N et al. *JAMA Network Open.*

eFigure 1. Flowchart of Study Cohorts

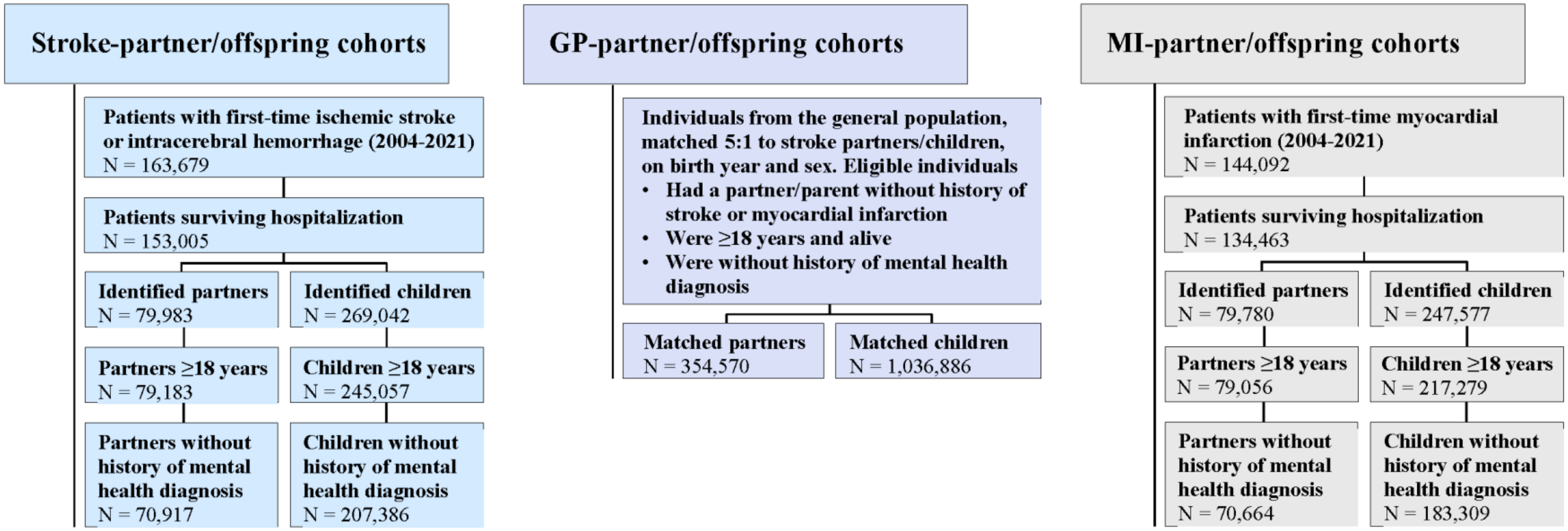

© 2024 Skajaa N et al. *JAMA Network Open.*

eFigure 2. Directed Acyclic Graph Depicting Causal Assumptions in This Study

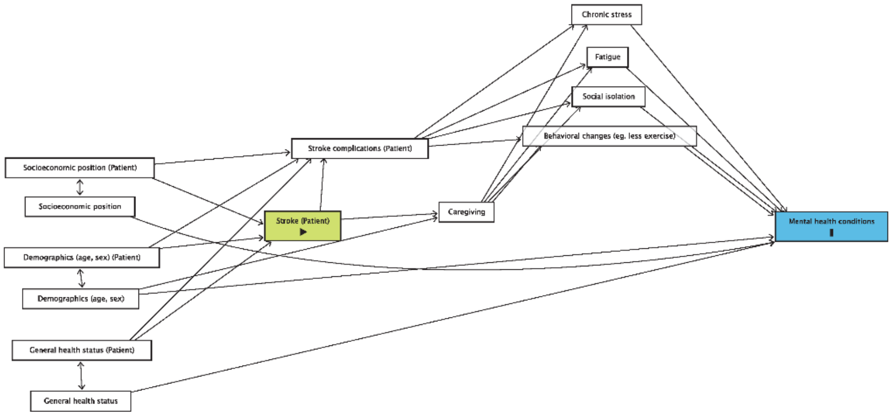

© 2024 Skajaa N et al. *JAMA Network Open.*

eFigure 3. Propensity Score Weighted Cumulative Incidences, 3-Year Risk Differences, and 3-Year Risk Ratios of Self-Harm or Suicide and Any Diagnosis of a Mental Health Condition Among Partners of Stroke Survivors (Stroke-Partner Cohort), Partners of Individuals From the General Population (GP-Partner Cohort), and Partners of Myocardial Infarction Survivors (MI-Partner Cohort)

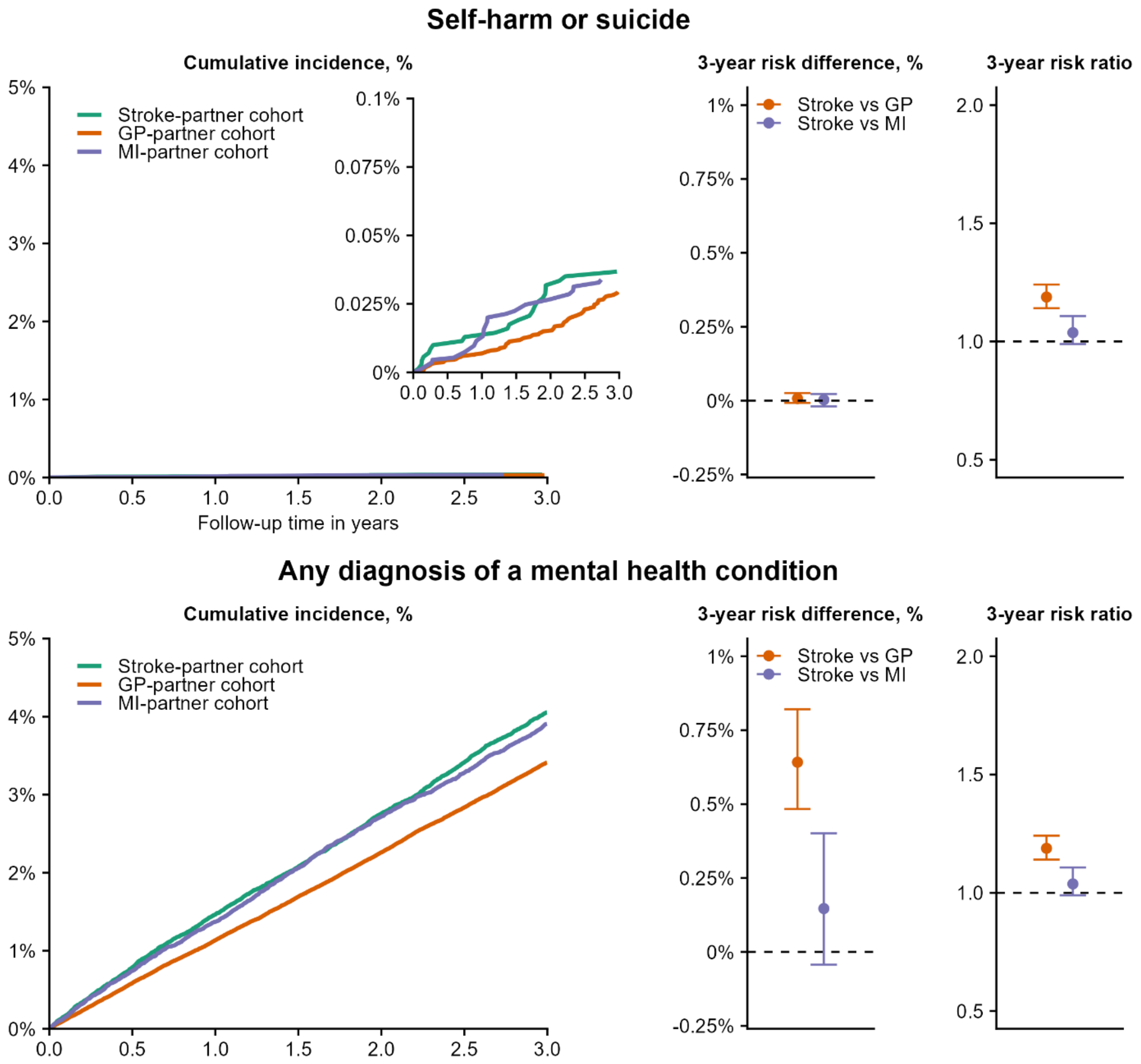

© 2024 Skajaa N et al. *JAMA Network Open.*

eFigure 4. Propensity Score Weighted Cumulative Incidences, 3-Year Risk Differences, and 3-Year Risk Ratios of Self-Harm or Suicide and Any Diagnosis of a Mental Health Condition Among Adult Children of Stroke Survivors (Stroke-Offspring Cohort), Adult Children of Individuals From the General Population (GP-Offspring Cohort), and Adult Children of Myocardial Infarction Survivors (MI-Offspring Cohort)

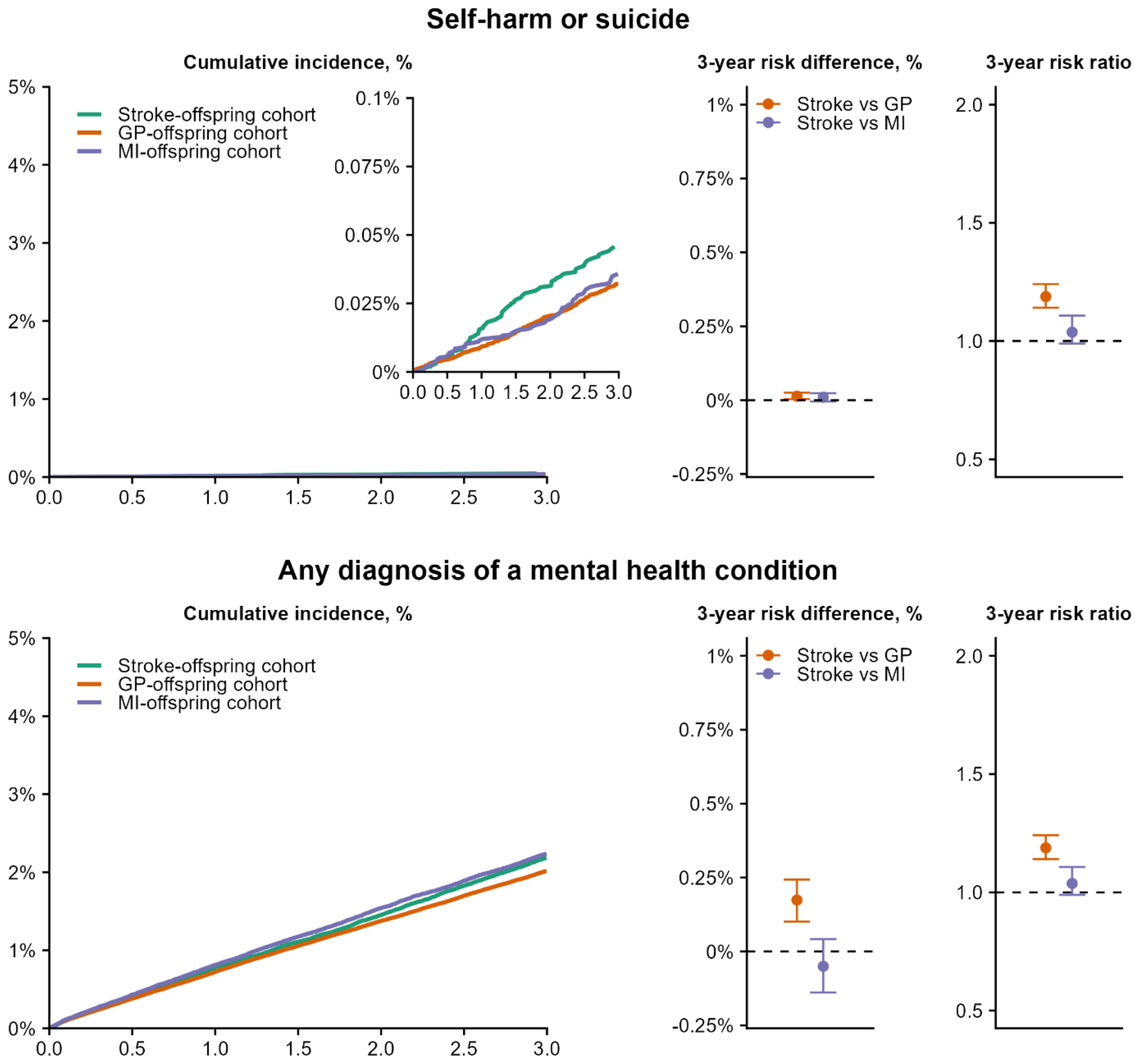

© 2024 Skajaa N et al. *JAMA Network Open.*

eReferences

1. Schmidt M, Pedersen L, Sørensen HT. The Danish Civil Registration System as a tool in epidemiology. *Eur J Epidemiol*. 2014;29(8):541-549. doi:10.1007/s10654-014-9930-3

2. Johnsen SP, Ingeman A, Hundborg HH, Schaarup SZ, Gyllenborg J. The Danish Stroke Registry. *Clin Epidemiol*. 2016;8:697-702. doi:10.2147/CLEP.S103662

3. Wildenschild C, Mehnert F, Thomsen RW, et al. Registration of acute stroke: validity in the Danish Stroke Registry and the Danish National Registry of Patients. *Clin Epidemiol*. 2013;6:27-36. doi:10.2147/CLEP.S50449

4. Hald SM, Kring Sloth C, Hey SM, et al. Intracerebral hemorrhage: positive predictive value of diagnosis codes in two nationwide Danish registries. *Clin Epidemiol*. 2018;10:941-948. doi:10.2147/CLEP.S167576

5. Schmidt M, Schmidt SAJ, Sandegaard JL, Ehrenstein V, Pedersen L, Sørensen HT. The Danish National Patient Registry: a review of content, data quality, and research potential. *Clin Epidemiol*. 2015;7:449-490. doi:10.2147/CLEP.S91125

6. Mors O, Perto GP, Mortensen PB. The Danish Psychiatric Central Research Register. *Scand J Public Health*. 2011;39(7 Suppl):54-57. doi:10.1177/1403494810395825

7. Pottegård A, Schmidt SAJ, Wallach-Kildemoes H, Sørensen HT, Hallas J, Schmidt M. Data Resource Profile: The Danish National Prescription Registry. *Int J Epidemiol*. 2017;46(3):798-798f. doi:10.1093/ije/dyw213

8. Helweg-Larsen K. The Danish Register of Causes of Death. *Scand J Public Health*. 2011;39(7 Suppl):26-29. doi:10.1177/1403494811399958

9. Baadsgaard M, Quitzau J. Danish registers on personal income and transfer payments. *Scand J Public Health*. 2011;39(7 Suppl):103-105. doi:10.1177/1403494811405098

10. Jensen VM, Rasmussen AW. Danish Education Registers. *Scand J Public Health*. 2011;39(7 Suppl):91-94. doi:10.1177/1403494810394715

11. Mainz J, Andersen G, Valentin JB, Gude MF, Johnsen SP. Treatment Delays and Chance of Reperfusion Therapy in Patients with Acute Stroke: A Danish Nationwide Study. *Cerebrovasc Dis*. 2023;52(3):275-282. doi:10.1159/000526733

12. Govan L, Langhorne P, Weir CJ. Categorizing stroke prognosis using different stroke scales. *Stroke*. 2009;40(10):3396-3399. doi:10.1161/STROKEAHA.109.557645

13. The Danish Stroke Association. *Action Plan for Stroke*.; 2022. https://www.hjernesagen.dk/wp-content/uploads/2022/02/ENDELIG_handleplan-for-stroke-2022-enkeltsider_med-links.pdf

14. Heide-Jørgensen U, Adelborg K, Kahlert J, Sørensen HT, Pedersen L. Sampling strategies for selecting general population comparison cohorts. *Clin Epidemiol*. 2018;10:1325-1337. doi:10.2147/CLEP.S164456

© 2024 Skajaa N et al. *JAMA Network Open.*

15. Hjorth CF, Damkier P, Ejlertsen B, Lash T, Sørensen HT, Cronin-Fenton D. Socioeconomic position and prognosis in premenopausal breast cancer: a population-based cohort study in Denmark. *BMC Med*. 2021;19(1):235. doi:10.1186/s12916-021-02108-z

16. Schmidt SAJ, Mailhac A, Darvalics B, et al. Association Between Atopic Dermatitis and Educational Attainment in Denmark. *JAMA Dermatol*. 2021;157(6):1-9. doi:10.1001/jamadermatol.2021.0009

17. Prior A, Fenger-Grøn M, Larsen KK, et al. The Association Between Perceived Stress and Mortality Among People With Multimorbidity: A Prospective Population-Based Cohort Study. *Am J Epidemiol*. 2016;184(3):199-210. doi:10.1093/aje/kwv324

18. Momen NC, Plana-Ripoll O, Agerbo E, et al. Association between Mental Disorders and Subsequent Medical Conditions. *N Engl J Med*. 2020;382(18):1721-1731. doi:10.1056/NEJMoa1915784

19. Skajaa N, Adelborg K, Horváth-Puhó E, et al. Labour market participation and retirement after stroke in Denmark: registry based cohort study. *BMJ*. 2023;380:e072308. doi:10.1136/bmj-2022-072308

20. Desai RJ, Franklin JM. Alternative approaches for confounding adjustment in observational studies using weighting based on the propensity score: a primer for practitioners. *BMJ*. 2019;367:l5657. doi:10.1136/bmj.l5657

21. Choi J, Dekkers OM, le Cessie S. A comparison of different methods to handle missing data in the context of propensity score analysis. *Eur J Epidemiol*. 2019;34(1):23-36. doi:10.1007/s10654-018-0447-z

22. Austin PC. Balance diagnostics for comparing the distribution of baseline covariates between treatment groups in propensity-score matched samples. *Stat Med*. 2009;28(25):3083-3107. doi:10.1002/sim.3697

23. Andersen PK, Keiding N. Multi-state models for event history analysis. *Stat Methods Med Res*. 2002;11(2):91-115. doi:10.1191/0962280202SM276ra

24. Puth MT, Neuhäuser M, Ruxton GD. On the variety of methods for calculating confidence intervals by bootstrapping. *J Anim Ecol*. 2015;84(4):892-897. doi:10.1111/1365-2656.12382

25. Izem R, Liao J, Hu M, et al. Comparison of propensity score methods for pre-specified subgroup analysis with survival data. *J Biopharm Stat*. 2020;30(4):734-751. doi:10.1080/10543406.2020.1730868

26. Lipsitch M, Tchetgen Tchetgen E, Cohen T. Negative controls: a tool for detecting confounding and bias in observational studies. *Epidemiology*. 2010;21(3):383-388. doi:10.1097/EDE.0b013e3181d61eeb

27. Odenbro A, Bellocco R, Boffetta P, Lindelöf B, Adami J. Tobacco smoking, snuff dipping and the risk of cutaneous squamous cell carcinoma: a nationwide cohort study in Sweden. *Br J Cancer*. 2005;92(7):1326-1328. doi:10.1038/sj.bjc.6602475

28. Draper BM, Poulos CJ, Cole AM, Poulos RG, Ehrlich F. A comparison of caregivers for elderly stroke and dementia victims. *J Am Geriatr Soc*. 1992;40(9):896-901. doi:10.1111/j.1532-5415.1992.tb01986.x

© 2024 Skajaa N et al. *JAMA Network Open.*

29. Anderson CS, Linto J, Stewart-Wynne EG. A population-based assessment of the impact and burden of caregiving for long-term stroke survivors. *Stroke*. 1995;26(5):843-849. doi:10.1161/01.str.26.5.843

30. Berg A, Palomäki H, Lönnqvist J, Lehtihalmes M, Kaste M. Depression among caregivers of stroke survivors. *Stroke*. 2005;36(3):639-643. doi:10.1161/01.STR.0000155690.04697.c0

31. Draper P, Brocklehurst H. The impact of stroke on the well-being of the patient's spouse: an exploratory study. *J Clin Nurs*. 2007;16(2):264-271. doi:10.1111/j.1365-2702.2006.01575.x

32. Rigby H, Gubitz G, Phillips S. A systematic review of caregiver burden following stroke. *Int J Stroke*. 2009;4(4):285-292. doi:10.1111/j.1747-4949.2009.00289.x

33. Rigby H, Gubitz G, Eskes G, et al. Caring for stroke survivors: baseline and 1-year determinants of caregiver burden. *Int J Stroke*. 2009;4(3):152-158. doi:10.1111/j.1747-4949.2009.00287.x

34. Cameron JI, Cheung AM, Streiner DL, Coyte PC, Stewart DE. Stroke survivor depressive symptoms are associated with family caregiver depression during the first 2 years poststroke. *Stroke*. 2011;42(2):302-306. doi:10.1161/STROKEAHA.110.597963

35. Haley WE, Roth DL, Hovater M, Clay OJ. Long-term impact of stroke on family caregiver well-being: a population-based case-control study. *Neurology*. 2015;84(13):1323-1329. doi:10.1212/WNL.0000000000001418

36. Loh AZ, Tan JS, Zhang MW, Ho RC. The Global Prevalence of Anxiety and Depressive Symptoms Among Caregivers of Stroke Survivors. *J Am Med Dir Assoc*. 2017;18(2):111-116. doi:10.1016/j.jamda.2016.08.014

© 2024 Skajaa N et al. *JAMA Network Open.*
