# Supplementary material for: Mental Health Conditions in Partners and Adult Children of Stroke Survivors
Source: JAMA Netw Open. 2024 Mar 14;7(3):e243286. doi: 10.1001/jamanetworkopen.2024.3286 (PMC10940969; doi:10.1001/jamanetworkopen.2024.3286)
Supplement: Supplement 2. — Data Sharing Statement [file jamanetwopen-e243286-s002.pdf]

Data Sharing Statement

Skajaa. Mental Health Conditions in Partners and Adult Children of Stroke Survivors. *JAMA Netw Open*. Published March 14, 2024. doi:10.1001/jamanetworkopen.2024.3286

Data
Data available: No
